# Stearoyl coenzyme A desaturase 1 (SCD1) regulates foot-and-mouth disease virus replication by modulating host cell lipid metabolism and viral protein 2C-mediated replication complex formation

**DOI:** 10.1128/jvi.00902-24

**Published:** 2024-09-26

**Authors:** Bonan Lv, Yuncong Yuan, Zhuang Yang, Xingran Wang, Jianjun Hu, Yidan Sun, Hang Du, Xuemei Liu, Huimin Duan, Ruyi Ding, Zishu Pan, Xiao-Feng Tang, Chao Shen

**Affiliations:** 1 Hubei Key Laboratory of Cell Homeostasis, College of Life Sciences, Wuhan University, Wuhan, China; 2 State Key Laboratory of Virology, College of Life Sciences, Wuhan University, Wuhan, China; University of Kentucky College of Medicine, Lexington, Kentucky, USA

**Keywords:** foot-and-mouth disease virus, SCD1, host lipid metabolism, oleic acid, replication complex

## Abstract

The life cycle of foot-and-mouth disease virus (FMDV) is tightly regulated by host cell lipid metabolism. In previous studies, we reported downregulated expression of stearoyl coenzyme A desaturase-1 (SCD1), a key enzyme of fatty acid metabolism, in BHK-VEC cells (a virus-negative cell line derived from BKH-21 cells with persistent FMDV infection) on comparing transcriptomic data for BHK-VEC and BHK-21 cells (Y. Yuan et al., Front Cell Infect Microbiol 12:940906, 2022, https://doi.org/10.3389/fcimb.2022.940906; L. Han et al., Vet Microbiol 263:109247, 2021, https://doi.org/10.1016/j.vetmic.2021.109247). In the present study, we identify that SCD1 regulates FMDV replication. SCD1 overexpression or exogenous addition of oleic acid (OA), a product of the enzymatic activity of SCD1, increased FMDV replication in both BHK-21 cells and SCD1-knockdown cells. Overexpression of SCD1 or exogenous addition of OA restored FMDV infection and replication in BHK-VEC cells, and OA also promoted FMDV replication in BHK-21 cells with persistent FMDV infection. SCD1 recruited the nonstructural FMDV protein 2C to a detergent-resistant membrane located in the perinuclear region of cells to form replication complexes. Inhibiting SCD1 enzyme activity resulted in a significantly decreased number of FMDV replication complexes with abnormal morphology. Inhibition of SCD1 activity also effectively decreased the replication of other RNA viruses such as respiratory enteric orphan virus-3-176, poliovirus-1, enterovirus 71, and vesicular stomatitis virus. Our results demonstrate that SCD1, as a key host regulator of RNA virus replication, is a potential target for developing novel drugs against infections by RNA viruses.

**IMPORTANCE:**

Many positive-stranded RNA viruses, including foot-and-mouth disease virus (FMDV), alter host membranes and lipid metabolism to create a suitable microenvironment for their survival and replication within host cells. In FMDV-infected cells, the endoplasmic reticulum membrane is remodeled, forming vesicular structures that rely heavily on increased free fatty acids, thereby linking lipid metabolism to the FMDV replication complex. Nonstructural FMDV protein 2C is crucial for this complex, while host cell enzyme stearoyl coenzyme A desaturase 1 (SCD1) is vital for lipid metabolism. We found that FMDV infection alters SCD1 expression in host cells. Inhibiting SCD1 expression or its enzymatic activity markedly decreases FMDV replication, while supplementing oleic acid, a catalytic product of SCD1, regulates FMDV replication. Additionally, SCD1 forms part of the FMDV replication complex and helps recruit 2C to a detergent-resistant membrane. Our study provides insights into the pathogenesis of FMDV and a potential novel drug target against the virus.

## INTRODUCTION

Foot-and-mouth disease (FMD) is an acute, highly contagious viral disease caused by foot-and-mouth disease virus (FMDV), which infects even-toed ungulates (such as cattle, sheep, and goats), causing rapid disease onset and high mortality. FMDV is a positive-strand RNA virus of the family *Picornaviridae* and genus *Aphthovirus*. Recent studies have demonstrated that many positive-strand RNA virus infections are accompanied by changes in host intracellular lipid metabolism, including remodeling of the host cell endosomal system, decreased fatty acid oxidation, increased *ab initio* lipid synthesis, and increased fatty acid transport rate (1). Positive-strand RNA viruses display increased survival and replication efficacies in host cells by coordinating virus–host interactions to remodel host cell membranes and lipid metabolism and altering the microenvironment (2). Dengue virus (DENV), a *Flavivirus* member, inhibits the *ab initio* synthesis of lipids and disrupts the formation of transporter vesicles, which enhances viral infection (3). In DENV-infected cells, fatty acid synthase (FASN) is relocated to the DENV replication site where viral nonstructural protein 3 (NS3) colocalizes and interacts with FASN. Inhibition of FASN enzymatic activity inhibits DENV infection, indicating that DENV relies on the host cell fatty acid biosynthesis pathway to establish its replication complex (4–6). In cells infected with DENV, the rate of fatty acid synthesis was significantly increased compared with that in uninfected cells, and inhibition of lipid synthesis resulted in significantly decreased DENV replication (4–6). Stearoyl coenzyme A desaturase 1 (SCD1) catalyzes the biosynthesis of monounsaturated fatty acids from saturated fatty acid precursors, and both the gene expression and enzyme activity of SCD1 were significantly upregulated early in cells infected with DENV2. This finding suggested that the catalytic products of SCD1 play important roles in the life cycle of this virus.

In FMDV-infected cells, the endoplasmic reticulum membrane is remodeled, and many new vesicular structures appear in the cytoplasm. The formation of these vesicular structures requires the participation of a large quantity of free fatty acids. The vesicular structures also contain nonstructural proteins of FMDV origin and cytokines of host cell origin (7, 8). This suggests that host cell lipid metabolism is closely related to FMDV replication complex formation. In cells infected with several small RNA viruses, type III phosphatidylinositol 4 kinase catalyzes the substrate to produce high levels of PI4P (phosphatidylinositol 4-phosphate), which is subsequently converted to cholesterol in the replication complex (9). However, the roles of host factors involved in lipid metabolism-mediated regulation of FMDV replication or replication complex formation have not been reported.

Studies on the functions of *SCD1-4*, members of the SCD family, have primarily focused on SCD1. *SCD1-3* is present in the Syrian hamster (10). SCD1 is a delta-9 desaturase and a rate-limiting enzyme in the biosynthesis of monounsaturated fatty acids from saturated fatty acid precursors in organisms. The enzymatic products of SCD1 [e.g., oleic acid (OA), diacylglycerol, phospholipids, triglycerides, palmitoleic acid, and cholesterol esters] are involved in the formation of the hepatitis C virus (HCV) replication complex (11). On the basis of these observations, we hypothesized that SCD1 enzymatic activity may be crucial for FMDV replication. SCD1 contains four transmembrane helices (TM1-4) that form the stem of the mushroom-shaped protein and a cytosolic structural domain that forms the top of the mushroom. TM2 and TM4, which are longer than TM1 and TM3, protrude into the cytosolic region, providing three of the nine histidine residues that coordinate two metal ions (12, 13). OA increases fluidity and structural changes in the phospholipid membrane bilayer, including negative curvature of biological membranes, which is essential for the formation of closed vesicle-like structures in cells. SCD1, which converts stearic acid (SA) to OA, is a key enzyme in the intracellular synthesis of OA. A significant decrease in the number of intracellular lipid droplets and specialized double-membrane vesicles was observed via electron microscopy after treating Huh7 (human hepatoma) cells with an SCD1 inhibitor (14). However, the effect of lipid metabolism, including OA, on viral replication has rarely been studied.

Here, we conducted an in-depth investigation of the role of SCD1-regulated lipid metabolism in FMDV replication and the underlying molecular mechanisms. Our findings reveal that SCD1 regulates FMDV replication by modulating host cell lipid metabolism and facilitating FMDV nonstructural protein 2C-mediated viral replication complex formation. Additionally, we discovered that SCD1 affects the replication of other RNA viruses such as respiratory enteric orphan virus-3-176 (REO176), poliovirus-1 (PV1), enterovirus 71 (EV71), and vesicular stomatitis virus (VSV). These results provide new insights for developing innovative strategies against RNA viruses.

## MATERIALS AND METHODS

### Cell lines and viruses

FMDV type O (Akesu/58/2002) was kindly provided by the Lanzhou Veterinary Research Institute, Chinese Academy of Agricultural Sciences. The FMDV titer was calculated using a plaque assay. Golden hamster kidney fibroblast cell lines BHK-21, BHK-Op (BHK-21 cells with persistent FMDV infection), and BHK-VEC (virus-negative cells isolated from among BHK-Op cells) were obtained from the Chinese Type Culture Center (CCTCC). PK-15 and Vero cells, PV1 (GDV057), EV71 (GDV067), REO176 (GDV070), and VSV (GDV027) were donated by the CCTCC.

### Cell culture

BHK-21 cells, BHK-Op cells, and BHK-VEC cells were cultured in T25 culture flasks containing 4–6 mL of minimum essential medium (MEM, Thermo Fisher) containing 10% fetal bovine serum (FBS, Every green) and 1% streptomycin and penicillin. All cells were cultured at 37°C in the presence of 5% CO_2_.

The SCD1-specific inhibitor (MK8245) was dissolved in dimethylsulfoxide (DMSO) or and stored at −20°C, the chemical formula of MK8245 (MCE) is C17H16BrFN6O4. The fatty acids used included oleic acid OA (18:1), and stearic acid SA (18:0). For example, cells were cultured in 12-well plates, and once they reached 60%–70% confluence, they were infected with FMDV [50% tissue culture infectious dose (TCID_50_) = 1 × 10^−4^ /mL], that is, the multiplicity of infection = 0.2. One hour later, the medium was replaced with MEM containing 2% FBS, followed by the addition of fatty acids or the indicated concentration of SCD1 inhibitor. The cells were collected at 16 or 24 h post-infection (hpi), and the RNA and protein levels were analyzed as previously described below.

### Plasmids and DNA transfection

To construct the overexpression vector we first isolated total RNA from BHK-21 cells and then reverse transcribed it to create cDNA. The cDNA was then used as a template for amplification of the desired gene fragment using Phanta Max Super-Fidelity DNA Polymerase (Vazyme). The amplified target fragment was subcloned and inserted into the pHAGE-CMV-MCS-IZsGreen (Thermo Fisher) vector using the ClonExpress II One Step Cloning Kit (Vazyme). Successful construction of the plasmid was verified by enzymatic digestion and DNA sequencing.

The knockdown plasmid for SCD1 was constructed by first designing and synthesizing a 21-residue oligonucleotide and annealing it to form a double-stranded short interfering RNA (siRNA). The SCD1 siRNA sequences that we used were Si-SCD#1: UGAGAGAAGAAAAAGCCACGG, Si-SCD#2: UGUUUUGCGCACAAGCAGCCAG, and Si-SCD#3: ACUGUUCACCAGCCAGGUGGC. The annealed double-stranded oligonucleotides were cloned between the *Hin*dIII and *Bgl*II restriction sites of the pSUPER.retro.puro plasmid (Oligoengine). The successful construction of the knockdown plasmid was verified by digestion using the restriction endonucleases *Eco*RI and *Hin*dIII.

Cells were cultivated in a 12-well plate at 37°C. When the cell density reaches about 60%, prepare transfection reagents A [1.6 µg plasmid + 100 µL opti-MEM (Thermo Fisher) per well] and B (1.6 µL lipofectamine 2000 + 100 µL opti-MEM per well) were prepared. Solutions A and B were mixed and added to the cells. The solution was changed 4–6 h later.

### Cytotoxicity assays

The drugs to be tested were MK8245, OA (C18H34O2), and SA (C18H36O2); they were all purchased from MCE. Eight concentrations of each drug to be tested (diluted with DMSO) were selected, and eight replicates were used for each concentration. Drugs were added to the cells to be tested, which were incubated at 37°C under 5% CO2. After 24 h, Cell Counting Kit-8 (CCK-8) reagent (Yeasen Biotech) was added (10% vol:vol), and the plates were subsequently incubated at 37°C in the presence of 5% CO2 for 2 h. Absorbance values were measured at 450 nm using the microplate reader (Thermo Fisher). The cell survival rate was calculated as follows: cell survival rate = (absorbance value of experimental group − absorbance value of blank control group)/(absorbance value of solvent group − absorbance value of blank control group).

### RNA extraction and reverse transcription quantitative PCR

The medium was removed from a 12-well plate and 500 µL of RNAiso Plus (TAKARA) was added to each well. The mixture was then incubated at 4°C for 30 min. Cells in each sample were extracted with 100 µL of chloroform and collected into RNase-free Eppendorf (EP) tubes. RNA was extracted from the cells by isopropanol precipitation.

cDNA was synthesized by reverse transcription of the RNA using a Hifair II 1st Strand cDNA Synthesis Kit (Yeasen), followed by chain-specific reverse transcription quantitative PCR (RT-qPCR) using SYBR Green dye and a CFX96 real-time PCR detection system. The expression level of *GAPDH* was used as an internal reference, and relative changes in the expression of target genes were calculated via the 2^−△△CT^ method. The primers for RT-PCR are listed in Table 1. GAPDH was used as an internal reference gene.

**TABLE 1:** Primers used in the research

Genes	Species	Sequences
*GAPDH*	Golden hamster	F: AAGGCCATCACCATCTTCCA
		R: GCCAGTAGACTCCACAACATAC
*FMDV 3D*	Golden hamster	F: GAACACATTCTTTACACCAGGAT
		R: CATATCTTTGCCAATCAACATCAG
*SCD1*	Golden hamster	F: GAAGACCGTTCCCCTCTACC
		R: TAGAGCTTGCAGGACGGAAC
*SCD2*	Golden hamster	F: TGTCTGGCTCTTACTCAACCA
		R: CAGGACGGAACCAGTGTGAT
*SCD3*	Golden hamster	F: CGCTGGCACATCAACTTCAC
		R: ACTCAGATGACCCCAGACTCA
*SCD*	Golden hamster	F: ACCACATTCTTCATCGACTGC
		R: CTCCAGTTCTTTTAATCCTGGC
*Acsl6*	Golden hamster	F: GGTCGATGCAGAGGAACTCA
		R: TGTGCTACAGGTTCACTCCG
*Acsl1*	Golden hamster	F: TGATCATCGAGCAGGGATGC
		R: ACATATGGGCGAGAGGCAAA
*Acsl3*	Golden hamster	F: GTCGGAGCTCACCATTCTTG
		R: AGCCAAACTGTCCAAGCTGT
*Acat1*	Golden hamster	F: GATCCTTCATGGGCAGCCTT
		R: CCCCTTGGATGACATTCCCC
*Acsbg1*	Golden hamster	F: CACTCAAGGCCAACCTCTCA
		R: AGTATAGGGAAGCTGCGTGC
*Acox3*	Golden hamster	F: CATACAGCCCTGCTCTACCG
		R: TCTTTCAGCTGCATACACAGT
*Hadha*	Golden hamster	F: GCTCCGGAAGTACGAGTCTG
		R: CTGCCAGGTCTGGGTTAGTG
*Hadhb*	Golden hamster	F: CGGTCCAGTGTTCCAAAGGA
		R: CCACGACGACATCACACTGA
*Acadm*	Golden hamster	F: GCAATGGGGGCTTTTGACAG
		R: GCGAGTTCAACCTTCATCGC
*Fads1*	Golden hamster	F: GCCTGACGCTCCCCTAAC
		R: GAGGCCCTTGTTGATGTGGA
*Fads2*	Golden hamster	F: CTTCCGCTGGGAGGAGATTC
		R: GCTTCAAGAACTTGCCCACG
*Acads*	Golden hamster	F: AGTGCCTTTCTTCACCCGAG
		R: TCGGGCACAGGTGCTTTATT
*EV71*	Golden hamster	F: GCTCTATAGGAGATAGTGTGAGTAGGG
		R: ATGACTGCTCACCTGCGTGTT
*REO176*	Golden hamster	F: CTGGAATCGTTCGGAGTA
		R: GGTGGCAATAAGCAATAAA
*PV1*	Golden hamster	F: TCCGGCCCCTGAATGCGGCT
		R: TGTCACCATAAGCAGCC
*VSV*	Golden hamster	F: TAATTCCACGAAGCACCGAG
		R: AATGAGCAACAGTCAAGGCA

### Western blotting

Sodium dodecyl sulfate (SDS) loading buffer [containing RIPA Strong Lysis Buffer (Beyotime)], was added to a 12-well plate to lyse the cells. The protein lysate was collected in an EP tube, heated at 100°C for 10 min in a dry heat block, and subsequently centrifuged at 12,000 rpm for 5 min. Samples were separated via 10% or 15% SDS‒polyacrylamide gel electrophoresis and subsequently transferred to polyvinylidene fluoride membranes. The membranes were blocked with 5% skim milk for 1–2 h and then incubated with a specific primary antibody (diluted with antibody diluent) at 4°C overnight, followed by washing three times with TBST (TBS+Tween) and incubating with a secondary antibody (horseradish peroxidase-conjugated goat anti-rabbit or goat anti-mouse antibody) for 2 h at room temperature. The proteins were subsequently visualized with an enhanced chemiluminescence reagent. Antibodies used in this experiment are listed in Table 2.

**TABLE 2:** Antibodies used in the research

Antibody	Company	Dilution ratio
GAPDH	Affinity	1:2,000
FMDV 3D	Abiocenter	1:2,000
SCD1	Affinity	1:2,000
Myc	ABclonal	1:2,000
Flag	Huabio	1:1,000
FMDV 2C	Huabio	1:2,000
FMDV dsRNA	English and Scientific Consulting Kft	1:200
AMPK	Huabio	1:1,000
p-AMPK	Huabio	1:1,000
Cav-2	ABclonal	1:1,000
Calnexin	Huabio	1:1,000
Goat anti-rabbit IgG	ABclonal	1:10,000
Goat anti-mouse IgG	ABclonal	1:10,000
SREBP1	Huabio	1:1,000

### Viral titer assay

When the confluence of the BHK-21 cells in the T25 culture flasks reached 80%–90%, the cells were digested with trypsin for passaging in 96-well plates. The viral stock solution to be tested was diluted with a gradient series of serum-free MEM. A diluted viral solution (100 µL) was added to each well (eight replicate wells for each concentration), and in the control group, the same volume of virus-free medium was added. A maintenance medium (100 µL) containing 2% FBS was added to each well, and the 96-well plate was incubated at 37°C under 5% CO_2_. Every 24 h, the cells in each well were observed under a microscope for cytopathic effects (CPE), and the number of wells in which CPE was observed at each dilution was counted for 3 days.

### Coimmunoprecipitation

Each flask (T25) was aspirated and washed twice with phosphate-buffered saline (PBS) and 1 mL of IP Lysis Mixture (Beyotime; with protease inhibitor cocktail and phenylmethylsulfonyl fluoride preadded); then, the flasks were incubated for 10 min at 4°C in a shaker. The samples were collected into EP tubes using a cell scraper. The antibodies were added to magnetic beads (MCE) treated with binding/washing buffer (diluted 1:2,000 with binding/washing buffer) and incubated overnight on a rotating mixer at 4°C. The samples (400 µL) were then incubated for 2 h on a rotating mixer at 4°C. Magnetic (MCE) separation was performed on a magnetic holder, and the samples were washed four times with binding/washing buffer. Finally, immunoprecipitated protein samples were prepared for subsequent immunoblotting using a denaturing elution method.

### Luciferase reporter assay

The promoter region of the *SCD1* target gene [1,000 bp upstream and downstream of the start codon, containing the classical serum response element (SRE)] was subcloned and inserted into a reporter vector containing the luciferase gene [pGL3-basic (15)]. The constructed pGL3-SRE reporter plasmid (100 ng) was cotransfected with phRL-TK (2 ng; internal reference, containing the sea kidney luciferase gene) into BHK-21 cells cultured in a 24-well plate for 24 h. Then, the 1× passive lysis buffer [50 mM Tris (pH 7.4), 150 mM NaCl, 1% Triton X-100, 1% sodium deoxycholate, 0.1% SDS] was added and the cells were completely lysed on a shaker for 20–30 min before collection. The intensity of the dual-luciferase fluorescence signal was detected using the Promega GLOMAX operating system (16).

### Immunofluorescence assay

Cells were cultured in 24-well plates, then they were fixed with 4% paraformaldehyde for 30 min. After fixation, the cells were washed three times with sterile PBS, permeabilized with 0.5% Triton X-100 (diluted in PBS) for 12 min, washed three more times with sterile PBS, and blocked with 5% bovine serum albumin (BSA; diluted in PBS) for 30 min after which the blocking solution was aspirated and discarded. Primary antibody (diluted with 1% BSA) was added to the cells, which were then incubated overnight at 4°C. The cells were washed three times with sterile PBST (PBS+Tween), the secondary antibody dilution was added, and the mixture was incubated at room temperature for 2 h. At the end of the incubation, the cells were washed three times with sterile PBST and observed under a fluorescence microscope (17).

### Transmission electron microscopy

Approximately one-third of the cell suspension was mixed with a 1:1 mixture of 0.1 M phosphate buffer and 2.5% glutaraldehyde, abandoning the supernatant. Glutaraldehyde (2.5%) was added for approximately 20 min to fix the cells, after which the samples were centrifuged at 2,000 rpm for 10 min. Afterward, the samples were vacuumed for 2–3 h using a vacuum device, and the cells were fixed for a second time. Where cells had formed larger clumps, they were cut into suitably sized clumps directly. The cut clumps were washed at least twice with 0.1 M phosphate buffer for approximately 10 min each time. The clumps were then fixed with 1% osmium tetroxide for 1–2 h at room temperature in the dark and then fixed for observation by transmission electron microscopy (TEM) (18).

### Detergent-soluble and detergent-resistant membrane preparation and flotation assay

Cells were lysed in TNE buffer (150 mM NaCl, 1.0% Triton X-100, 3 mM EDTA, and 20 mM Tris-HCl), and a mixture of phosphatase and protease inhibitors was added. The cell lysate was then homogenized 10 times by passage through a 23-gauge needle and incubated on ice for 1 h. The lysate was centrifuged at 14,000 × *g* at 4°C for 30 min, and the supernatant was collected as the detergent-soluble fraction. The remaining (precipitated) fraction was resuspended in the same lysis buffer supplemented with 0.5% SDS and 2 mM dithiothreitol and subjected to brief sonication, following which the supernatant was collected as the detergent-resistant fraction (19).

### Construction of knockout strains by the electrotransfer method

The sequence of the coding region of the SCD1 gene was searched, and six pairs of single guide RNA (sgRNA) oligonucleotide sequences were designed to target exons 4 and 6 of the gene according to the principle “20*N*+NGG” by using the target site prediction tool (https://portals.broadinstitute.org/gpp/public/); CACC was added at the 5′- end of the sense strand. The programmed annealed double-stranded sgRNAs were ligated to the purified recovered products by using T4 DNA ligase (Thermo Fisher), and then transformed into Trans1-T1 chemoreceptor cells, and the correct monoclonal plasmids were selected by PCR.

Afterward, 3 × 106 cells were placed in a sterile tube, resuspended in 600 µL PBS, 30 µg of endotoxin-free CRPSPR-U (Thermo Fisher) expression plasmid was added, and mixed well. Electrotransfer Solution (3 mL) was added to an Electrode Cup and placed into the slot of the Electrotransferometer (Thermo Fisher). The cell–plasmid mixture was aspirated using a 100 µL electro-transfer tip, inserted into the shock cup, and shocked at 200 V for 20 ms. After electrotransformation, the cells were inoculated into 6-well plates prepared with a pre-warmed medium for incubation. The selected monoclonal cell lines were subjected to PCR and western blotting. The obtained monoclonal cell lines without the target gene were amplified and used for subsequent experiments.

### Statistical analysis

Student’s *t* test was used for statistical analysis with GraphPad Prism version 8.0, ImageJ, and Image Lab 5.2 software. A *P* value < 0.05 was considered to indicate statistical significance. The data are expressed as the mean ± standard deviation.

## RESULTS

### SCD1 is an essential host factor for regulating FMDV replication

**Fig 1:**
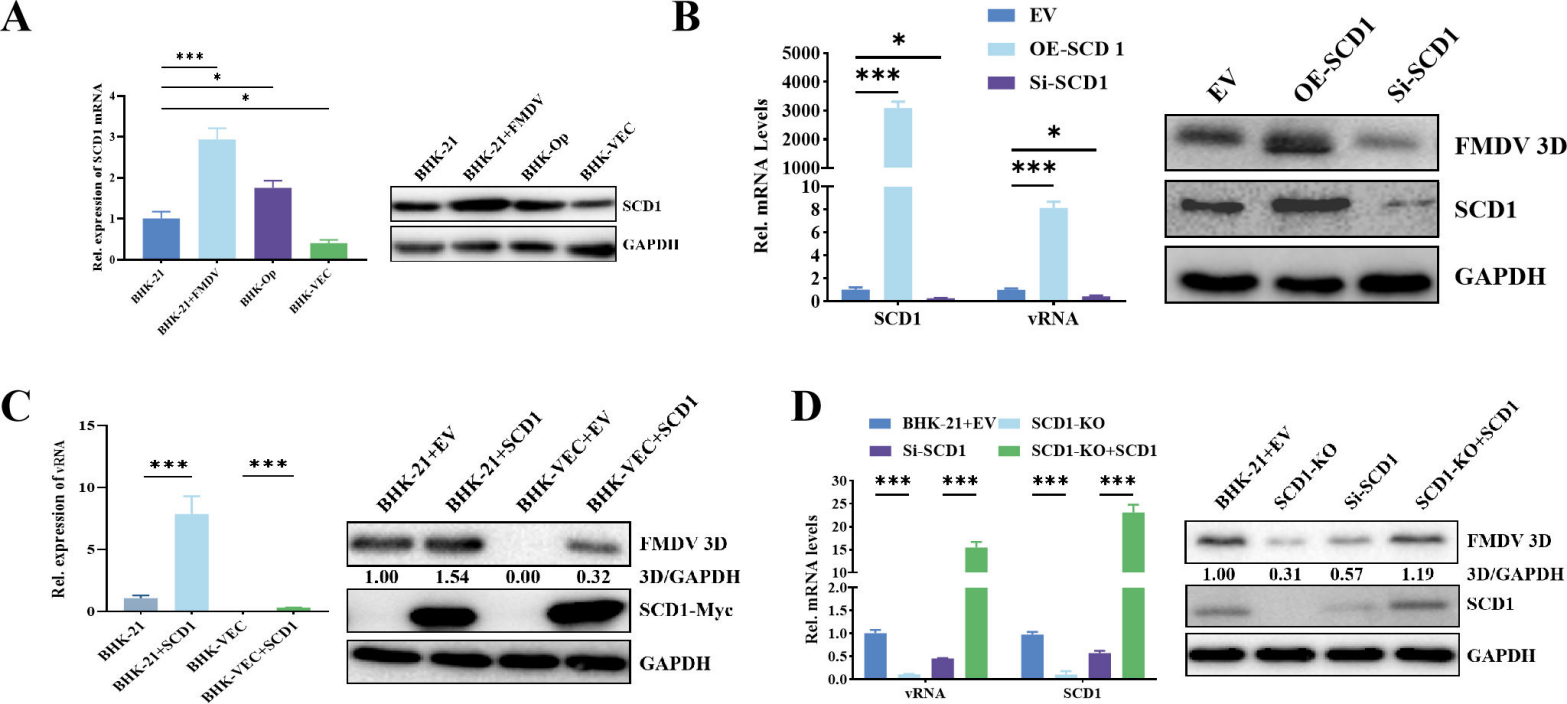
Identification of the function of SCD1 in regulating FMDV replication. (A) mRNA levels of *SCD1* mRNA levels in BHK-21 cells, BHK-21 cells infected with FMDV, BHK-VEC cells, and BHK-Op cells. (**B**) BHK-21 cells were transfected with a phage-CMV-IZsGreen (EV) SCD1-overexpression (OE-*SCD1*) plasmid or RNA interference plasmid (Si-*SCD1*) for 24 h and then infected with FMDV for 16 h. Detection of FMDV protein 3D and SCD1 expression by qPCR and western blotting. (**C**) BHK-VEC cells were transfected with the OE-*SCD1* plasmid for 24 h and then infected with FMDV for 16 h. Detection of 3D and SCD1 expression by qPCR and western blotting. (**D**) SCD1 mRNA and protein, vRNA, and FMDV 3D protein in the BHK-21-derived SCD1-knockout (KO) cell line were collected 16 h after FMDV infection. The protein bands were quantified in grayscale by using Image-Pro Plus 6.0 software and western blots. *n* = 3 for each group of experiments and three parallel samples were combined for western blotting. *P < 0.05; n.s., not significant.

We previously established the persistently FMDV-infected BHK cell line (BHK-Op) by single-cell selection (20), and an FMDV-negative cell clone (BHK-VEC) was isolated by single-cell clonal screening of high-passage BHK-Op cells (detoxified cells) (21). To understand the reason for the virus resistance of BHK-VEC cells, we analyzed differentially expressed genes (DEGs) between BHK-VEC and BHK-21 cells by transcriptomic analysis (20). DEGs were related to fatty acid metabolism pathways. Fifteen representative DEGs were selected for validation by RT-qPCR (Fig. S1A). *SCD1* mRNA levels were significantly downregulated in BHK-VEC cells compared with BHK-21 cells. *SCD1* expression was significantly upregulated in FMDV-infected BHK-21 cells compared with FMDV-uninfected BHK-21 cells (Fig. 1A).

To investigate the functions of the SCD family, we constructed RNAi plasmids targeting homologous regions in the *SCD1*, *SCD2*, and *SCD3* genes. These RNAi constructs effectively suppressed *SCD1*, *SCD2*, and *SCD3* expression, with the most significant inhibition observed for *SCD1* (Fig. S1B and C). Inhibition of *SCD1* expression in FMDV-infected BHK-21 cells led to a significant decrease in the levels of FMDV vRNA (Fig. S1B and C). Consistent with the mRNA level, the level of FMDV protein 3D was also significantly decreased in FMDV-infected cells following SCD1 inhibition (Fig. S1B and C). These results suggest that *SCD1* modulates FMDV replication in infected BHK-21 cells. We also investigated the effect of the overexpression of the other two SCD1 isoforms on FMDV infection. The data showed no significant difference in the protein levels of FMDV 3D for overexpressed SCD2 or SCD3 compared with the control group (Fig. S1D and E). These findings indicate that the suppression of FMDV infection in BHK-21 cells due to SCD gene inhibition is likely related to SCD1 rather than by its isoforms SCD2 and SCD3.

To investigate the regulatory effect of *SCD1* expression on FMDV replication, we overexpressed or knocked down SCD1 in BHK-21 cells. At 16 h post-transfection (hpt) with overexpression or knockdown constructs, the transfected cells were infected with FMDV, and samples were collected at 16 hpi. vRNA levels and FMDV 3D protein expression of FMDV were significantly upregulated in *SCD1-*overexpressing cells and significantly downregulated in *SCD1*-knockdown cells (Fig. 1B). Surprisingly, overexpression of SCD1 in BHK-VEC cells restored FMDV replication (Fig. 1C).

We used CRISPR (clustered regularly interspaced short palindromic repeats) technology to construct an SCD1-knockout (KO) cell line from BHK-21 cells. SCD1 KO significantly decreased the level of 3D protein compared with that in BHK-21 cells (Fig. 1D), indicating that the replication of FMDV was dependent on SCD1 expression. Overexpression of SCD1 in SCD1-KO cells (Fig. 1D) restored 3D protein expression. Our results demonstrate that SCD1 is an important host factor necessary for FMDV replication.

### SCD1 enzyme activity is essential for upregulating FMDV replication

**Fig 2:**
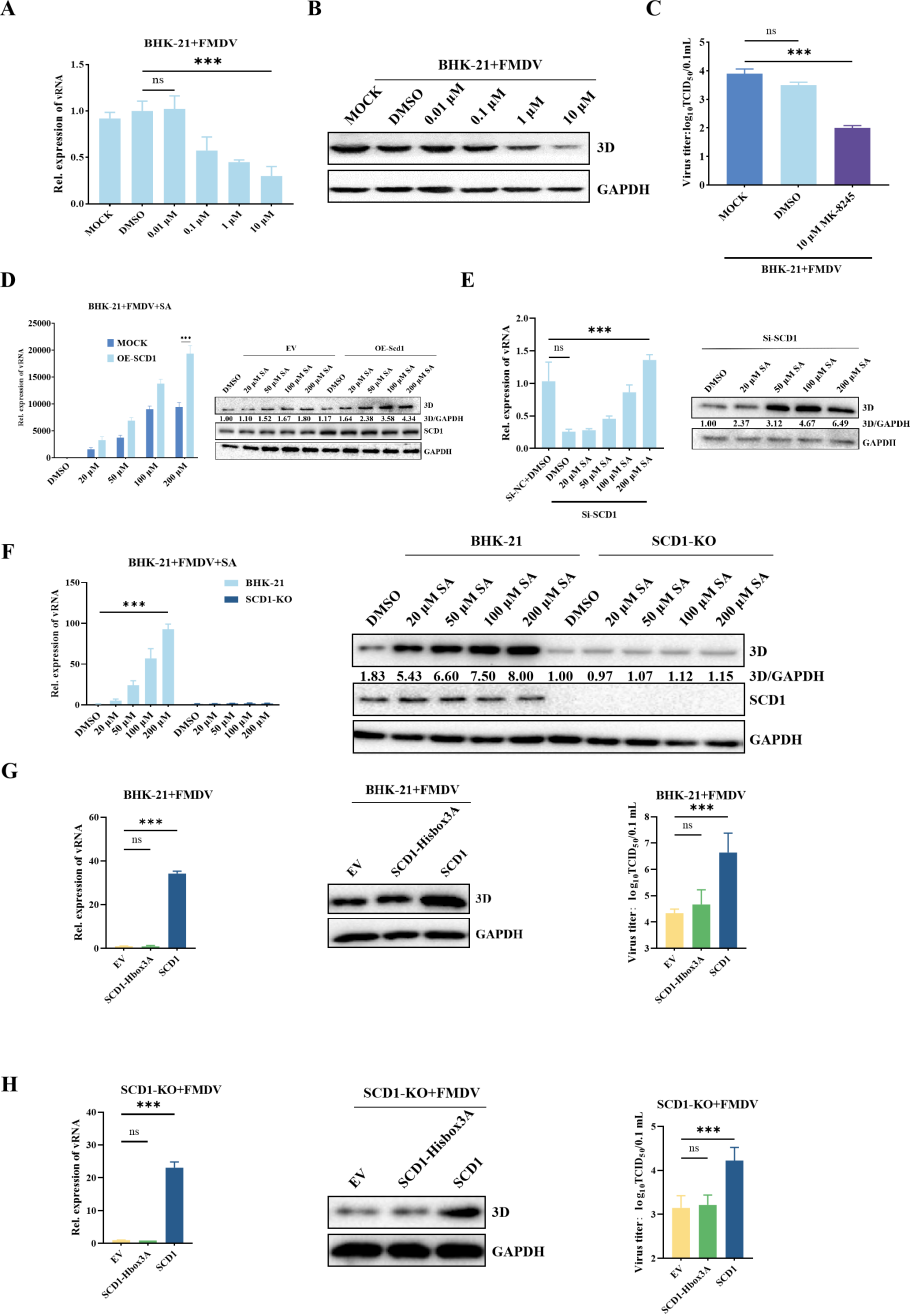
SCD1 enzyme activity is essential for promoting FMDV replication. (A) FMDV-infected BHK-21 cells were treated with a maintenance medium containing different concentrations of MK8245 for 1 h, after which the samples were collected after 16 h of culture. vRNA targeting 3D was analyzed by qPCR. (**B**) The 3D expression levels in A were analyzed by western blotting. (**C**) The viral titer in A was also determined by the TCID_50_ method. (**D**) FMDV-infected BHK-21 cells were treated with SA, and RNA was extracted and reverse-transcribed to cDNA at 16 hpi. BHK-21 cells were transfected with OE-*SCD1*, infected with FMDV, and treated with SA at 24 hpt. After 16 h of culture, the cells were harvested for vRNA and 3D protein detection. (**E**) BHK-21 cells were transfected with Si-*SCD1*, infected with FMDV, and treated with different concentrations of SA at 24 hpt. Cells were collected at 16 hpi for vRNA and 3D protein detection. (**F**) *SCD1*-KO BHK-21 cells or BHK-21 cells infected with FMDV were cultured in media containing different concentrations of stearic acid, after which the cells were collected for 3D expression analysis with GAPDH normalization. (**G**) The effect of overexpressing wild-type SCD1 or its mutant on 3D expression. BHK-21 cells were transfected with the indicated plasmids and infected with FMDV at 16 hpt. Cells were collected for vRNA and 3D protein expression analysis. The culture supernatant was harvested for virus titration using the TCID_50_ method. (**H**) The effect of overexpressing wild-type SCD1 or its mutant on 3D expression. *SCD1*-KO cells were transfected with the indicated plasmids and infected with FMDV at 16 hpt. Cells were collected for vRNA and 3D protein expression analysis. The culture supernatant was harvested for virus titration using the TCID_50_ method. The protein bands were quantified in grayscale by using Image-Pro Plus 6.0 software for the western blot. *n* = 3 for each group of experiments and three parallel samples were combined for western blotting. *n* = 3; *P < 0.05; n.s., not significant.

To investigate the role of SCD1 enzyme activity in regulating FMDV replication, we treated BHK-21 cells with MK8245, an inhibitor of SCD1 enzyme activity. We first assayed the cytotoxicity of MK8245 toward BHK-21 cells using a CCK-8 assay, and the IC_50_ value of MK8245 was ≥50 µM (Figure S2A and B). FMDV replication was significantly downregulated by MK8245 in a dose-dependent manner, and FMDV 3D protein expression was also significantly decreased (Fig. 2A and B). The addition of MK8245 significantly decreased the titer of FMDV (Fig. 2C). To elucidate the role of the SCD1 enzymatic product (unsaturated fatty acids) in FMDV replication, we evaluated the relationship between FMDV replication and unsaturated fatty acids by adding exogenous stearic acid (SA), a substrate of SCD1. The CC_50_ value for SA was ≥1,000 µM (Fig. S2C). Exogenous addition of SA promoted FMDV replication, and this effect was further enhanced by SCD1 overexpression, leading to increased FMDV 3D protein and RNA levels (Fig. 2D). After knockdown of SCD1, addition of exogenous SA significantly upregulated FMDV replication (Fig. 2E). However, in *SCD1*-KO cells, exogenous SA did not affect FMDV replication (Fig. 2F), suggesting that SCD1 enzymatically converts SA to OA, which facilitates FMDV replication. These data demonstrate that monounsaturated fatty acids, especially OA, the product of SCD1 enzymatic activity, are specifically required for FMDV replication.

The histidine cassette of SCD1 is involved in catalyzing the conversion of SA into unsaturated fatty acids (22, 23). To further confirm the role of SCD1 catalytic activity in regulating FMDV replication, we mutated the histidine residues at positions 297, 300, and 301 in SCD1 to alanine, to create the *SCD1*-Hbox3A mutant. We transfected these mutants into BHK-21 and *SCD1-KO* cells. Compared with cells transfected with SCD1, the FMDV 3D protein level was significantly lower in cells transfected with *SCD1*-Hbox3A (Fig. 2G and H), indicating that these histidine residues of SCD1 histidine residues are essential for its enzymatic activity.

### SCD1 catalytic product significantly upregulates FMDV replication in BHK-Op cells and triggers FMDV replication in BHK-VEC cells

**Fig 3:**
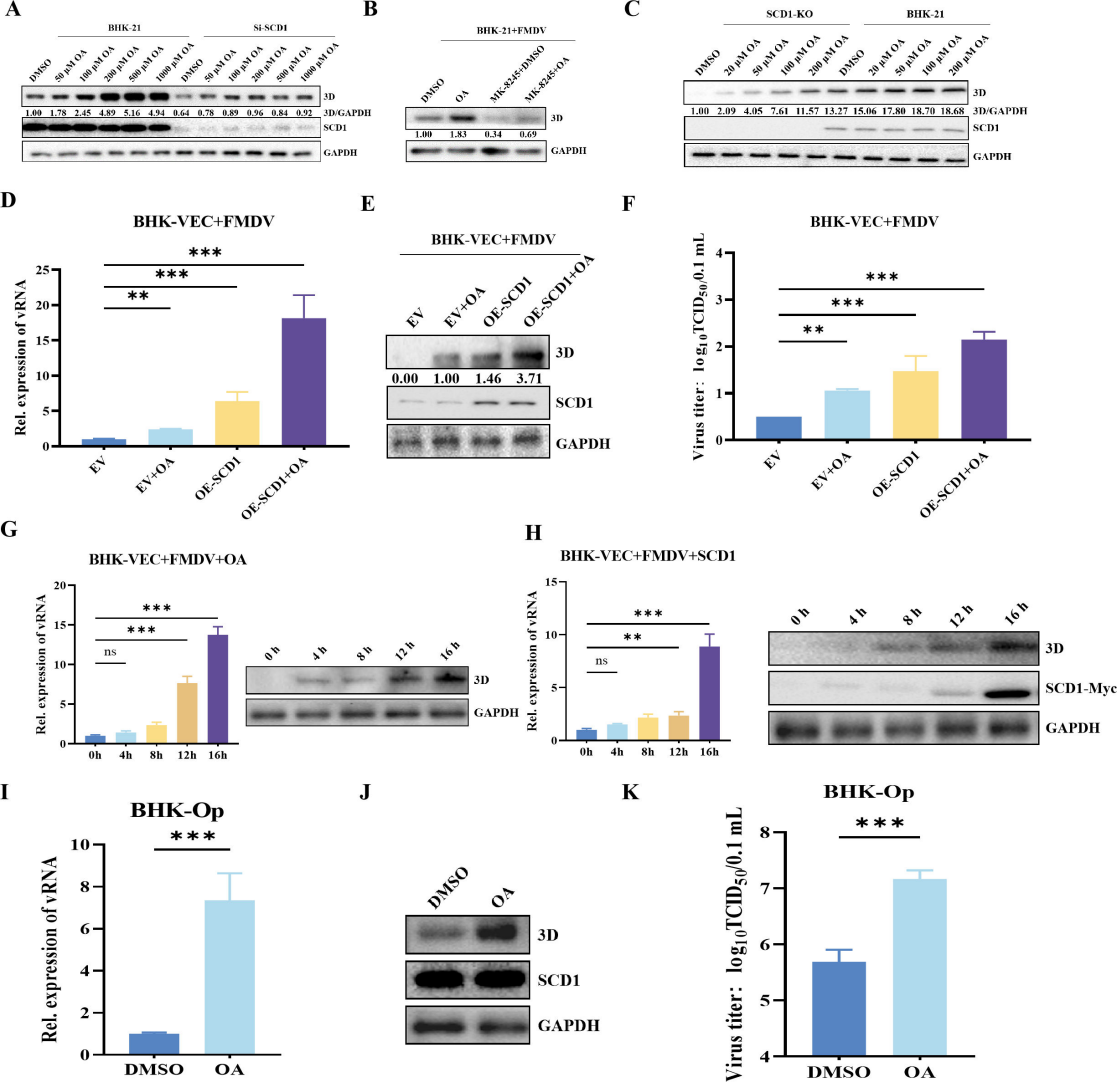
SCD1 enhances FMDV replicative efficiency in persistently infected cells and triggers FMDV replication in BHK-VEC cells. (A) BHK-21 cells transfected with Si-*SCD1* were infected with FMDV. At 24 hpi, the cells were cultured in media supplemented with different concentrations of OA. After 16 h of culture, the cells were harvested for analysis of expression of 3D. (**B**) FMDV-infected BHK-21 cells were treated with 20 µM MK8245 and 200 µM OA. After 16 h of culture, the cells were collected for analysis of FMDV protein 3D expression. (**C**) *SCD1*-KO BHK-21 cells or BHK-21 cells were infected with FMDV and cultured in media containing different concentrations of OA. After 16 h of culture, the cells were harvested for detection of 3D; *GAPDH* expression was used for normalization. (**D**) BHK-VEC cells transfected with OE-*SCD1* were infected with FMDV 16 hpt. Then, the infected cells were cultured in media supplemented with OA. After 16 h of culture, the cells were collected for the detection of vRNA. (**E**) The samples in panel (**D**) were also collected for the detection of 3D protein. (**F**) Viral titers of the samples in panel (**D**) were measured by the TCID_50_ method. (**G**) BHK-VEC cells were infected with FMDV and cultured in media containing OA for 16 h. Cells were collected at 0, 4, 8, 12, and 16 hpi for analysis of vRNA and 3D protein expression. (**H**) BHK-VEC cells were transfected with a plasmid expressing Myc-tagged SCD1. At 16 hpt, the cells were infected with FMDV and cultured in media containing OA for 16 h. Cells were collected at 0, 4, 8, 12, and 16 hpi for analysis of 3D and SCD1 mRNA and protein expression levels. (**I**) BHK-Op cells were cultured in media supplemented with OA for 16 h, after which the cells were collected for analysis of vRNA expression. (**J**) BHK-Op cells were cultured in media supplemented with OA for 16 h, after which the cells were collected for analysis of 3D protein expression. (**K**) Viral titers were measured by the TCID_50_ method after cell treatment. Protein bands were quantified in grayscale by using Image-Pro Plus 6.0 software and western blots. *n* = 3 for each group of experiments and three parallel samples were combined for western blotting. *P < 0.05; n.s., not significant.

To further whether the SCD1-mediated FMDV replication is dependent on unsaturated fatty acids, we examined the relationship between FMDV infection and exogenously added OA. Using a CCK-8 assay, we first verified that OA is not toxic to BHK-21 cells (Fig. S2D). Cells infected with FMDV and supplemented with different concentrations of OA were collected after 16 h of culture. Data from RT-qPCR and western blot analysis showed that the FMDV content in SCD1-KO BHK-21 cells was significantly lower than that in wild-type BHK-21 cells. Additionally, OA reversed the inhibition of FMDV replication caused by knockdown of *SCD1* in a dose-dependent manner (Fig. 3A). When the OA concentration reached 200 µM, the levels of FMDV 3D protein levels were not significantly different from those in the control. Thus, in subsequent experiments, we used 200 µM OA, which is far below the CC_50_ of OA. Addition of MK8245 to FMDV-infected BHK-21 cells inhibited SCD1 enzyme activity and thus FMDV replication; the addition of OA reversed this inhibition (Fig. 3B). Similarly, the addition of OA restored the inhibited FMDV replication in *SCD1*-KO cells (Fig. 3C).

We transfected BHK-VEC cells with an SCD1 overexpression plasmid, and at 24 hpt we infected the transfected cells with FMDV. The cells were harvested at 16 hpi for expression analysis. The data showed that overexpression of SCD1 activated FMDV replication in BHK-VEC cells (Fig. 3D through F). The exogenous addition of OA also activated FMDV replication in BHK-VEC cells. These data suggest that SCD1 activity is required for FMDV replication in BHK-VEC cells. To investigate the role of *SCD1* overexpression or exogenous OA in BHK-VEC cells, treated cell cultures were collected 0, 4, 8, 12, and 16 h after FMDV infection and analyzed by RT-qPCR and western blotting. In the OA-treated group, FMDV 3D protein expression was most significantly upregulated 12 hpi, while in the SCD1-overexpressing group, FMDV 3D protein expression was most significantly upregulated 16 hpi (Fig. 3G and H).

We also investigated the regulatory effect of exogenous OA on FMDV replication in BHK-Op cells. FMDV 3D expression and FMDV titer were significantly increased in BHK-Op cells after exogenous OA addition (Fig. 3I through K).

These results indicate that SCD1 regulates FMDV replication in BHK-Op cells and activates FMDV replication in BHK-VEC cells.

### SCD1 recruits FMDV 2C to detergent-resistant membranes to promote FMDV replication complex establishment

**Fig 4:**
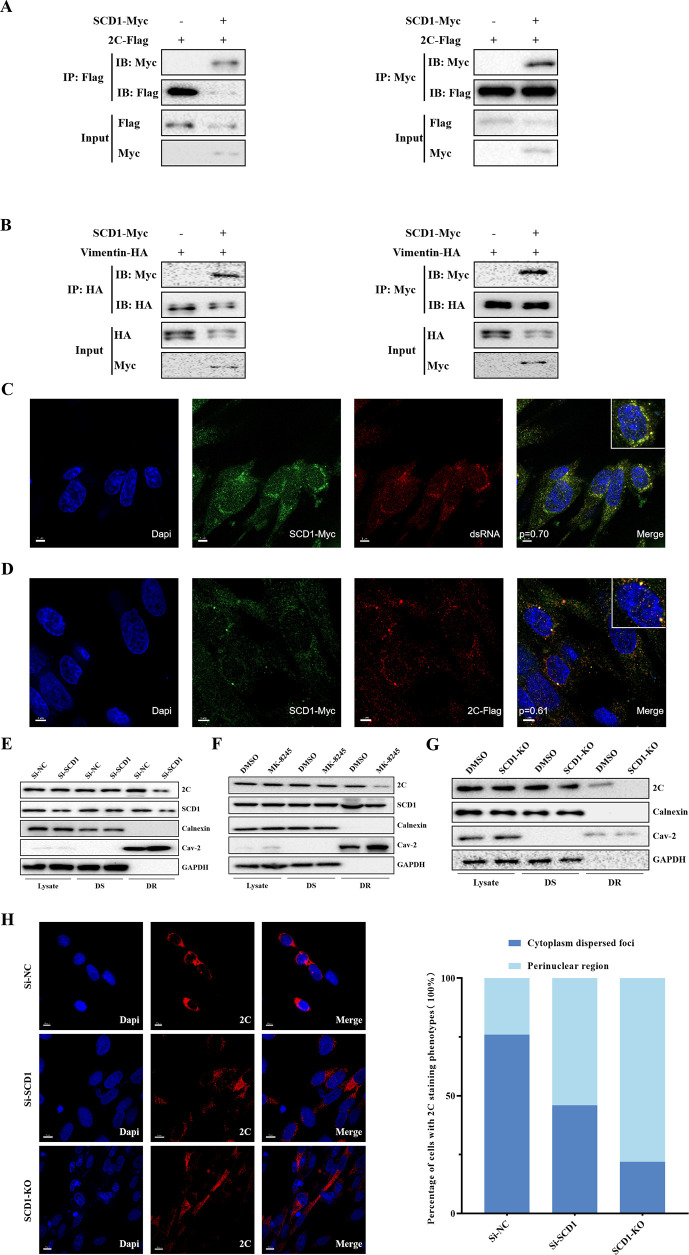
SCD1 recruits FMDV 2C to detergent-resistant membranes. (**A**) BHK-21 cells were co-transfected with plasmids expressing Myc-tagged SCD1 (SCD1-Myc) and Flag-tagged FMDV protein 2C (Flag-2C). The cells were harvested 24 hpt to detect the interaction between SCD1 and 2C in co-IP assays. (**B**) BHK-21 cells were co-transfected with plasmids expressing SCD1-Myc and HA-tagged vimentin (vimentin-HA). Cells were collected 24 hpt to detect the interaction between SCD1 and vimentin in co-IP assays. (**C**) BHK-21 cells were transfected with SCD1-Myc, and the cells were infected with FMDV 24 hpt. After 6 h of culture, the localization of SCD1 and double-stranded RNA (dsRNA) was analyzed using anti-SCD1 or dsRNA antibody staining. DAPI (4′,6-diamidino-2-phenylindole) staining was used to label nuclei (blue). (**D**) BHK-21 cells were co-transfected with plasmids expressing SCD1-Myc and Flag-2C, and the cells were infected with FMDV 24 hpt. After 6 h of culture, the localization of SCD1 and 2C was analyzed using antibodies directed against SCD1 and 2C by immunofluorescence analysis. DAPI staining was used to label nuclei (blue). The Pearson coefficients for panels (**C**) and (**D**) are labeled on the corresponding merged plots. (**E**) BHK-21 cells were transfected with SCD1-KO cells, and the cells were infected with FMDV 24 hpt. After 16 h of culture, the cells were collected and lysed, and the lysates were separated into detergent-soluble (DS) and detergent-resistant (DR) membrane fractions. Cell lysates and DS and DR membrane fractions were analyzed by immunoblotting with the indicated antibodies. (**F**) BHK-21 cells were infected with FMDV and cultured in a medium supplemented with an SCD1 inhibitor. After 16 h of culture, the cell lysates and DS and DR membrane fractions were analyzed. (**G**) SCD1-KO cells were infected with FMDV. At 16 hpi, the cells were harvested and lysed, and the cell lysates and DS and DR membrane fractions were analyzed. (**H**) Localization of FMDV protein 2C in SCD1-knockdown BHK-21 cells and SCD1-KO BHK-21 cells. For SCD1-knockdown, cells were transfected with Si-SCD1. At 24 hpt, the cells were infected with FMDV. After 16 h of culture, the subcellular localization of 2C was analyzed using an anti-2C antibody and immunofluorescence analysis. DAPI staining was used to label nuclei (blue; upper panel). Fifty 2C-positive cells were randomly selected for counting (expressed as a percentage). Three independent replicates were performed for each set of experiments. *n* = 3; *P < 0.05; n.s., not significant.

To understand the functional mechanism by which SCD1 mediates FMDV replication complex formation, we further investigated whether SCD1 interacts with the nonstructural protein 2C in the FMDV replication complex. Flag-tagged FMDV 2C and Myc-tagged SCD1 were cotransfected into BHK-21 cells. The cells were infected with FMDV at 24 hpt, and the infected cells were collected at 16 hpi. IP data (Fig. 4A) showed that SCD1 interacted with 2C, an FMDV replication complex protein that plays a key role in both membrane rearrangement and formation of the viral replication complex (24). Further study revealed that FMDV replication complexes contained FMDV 2C, vimentin, and SCD1. Coimmunoprecipitation (Co-IP) experiments (Fig. 4B) confirmed the interactions among these three proteins.

To further explore whether SCD1 is located within the FMDV replication complex, we used immunofluorescence (IF) to probe whether SCD1 colocalizes with the replication complex marker double-stranded RNA (dsRNA). We transfected a Myc-tag-SCD1 expressing plasmid into BHK-21 cells, and the transfected cells were infected with FMDV 24 hpt. We determined the colocalization of SCD1, 2C, and dsRNA components based on the Pearson coefficient: if the Pearson coefficient was >0.5, the two components were considered to be colocalized. The data showed Pearson’s coefficients of 0.70 for SCD1 with dsRNA, and 0.61 for SCD1 with 2C, both of which were greater than 0.5. We therefore concluded that SCD1 co-localizes with dsRNA and that 2C has the same localization pattern as SCD1 (Fig. 4C and D), suggesting that SCD1 is a constitutive protein of the FMDV replication complex.

The classical approach to studying plasma membrane microregions involves obtaining detergent-resistant extracted membrane fractions from cell membranes and analyzing their composition. We lysed *SCD1*-KO BHK-21 cells and SCD1 inhibitor-treated BHK-21 cells. Then, the cells were collected and divided into detergent-resistant and detergent-sensitive membrane fractions. Knockdown of *SCD1* significantly downregulated 2C in detergent-resistant membranes. Similarly, significantly decreased 2C concentration was observed in the detergent-resistant membranes of inhibitor-treated cells (Fig. 4E through G). In the inhibitor-treated group, the 2C concentration in the detergent-resistant membrane was also significantly reduced, probably due to the inhibition of SCD1 activity and consequent inability to recruit 2C. Our results demonstrate that SCD1 recruits 2C to detergent-resistant membranes.

To further investigate the membrane rearrangement induced by the 2C-mediated recruitment of SCD1, we observed the subcellular localization of 2C in *SCD1*-KO cells. Knockdown of SCD1 did not affect cell activity, and 2C protein expression was not altered by *SCD1* knockdown (Fig. S3A and B). After FMDV infection, 2C formed large and concentrated foci around the nucleus, suggesting that 2C was enriched in the region where replication complexes are concentrated (Fig. 4H). The distribution and subcellular localization of 2C in *SCD1*-KO cells revealed a dispersed pattern and increased disorder (Fig. 4H), suggesting that the recruitment of 2C is SCD1 dose-dependent. Statistical analysis of data from 100 randomly selected cells (Fig. 4H) showed that the intracellular distribution of 2C protein was diffuse after the reduction of SCD1 concentration, with 2C protein decreasing in the perinuclear region and becoming more abundant in the cytoplasm.

In conclusion, knockdown or KO of *SCD1* altered the subcellular localization of 2C. In the FDMV-infected *SCD1*-KO cell line, the localization of 2C was disrupted, which led to the downregulation of FMDV replication. We hypothesized that SCD1 recruits 2C and assists in the correct localization of 2C in the FMDV replication complex contributing to FMDV replication.

### SCD1 and its enzymatic products are involved in FMDV replication complex formation

**Fig 5:**
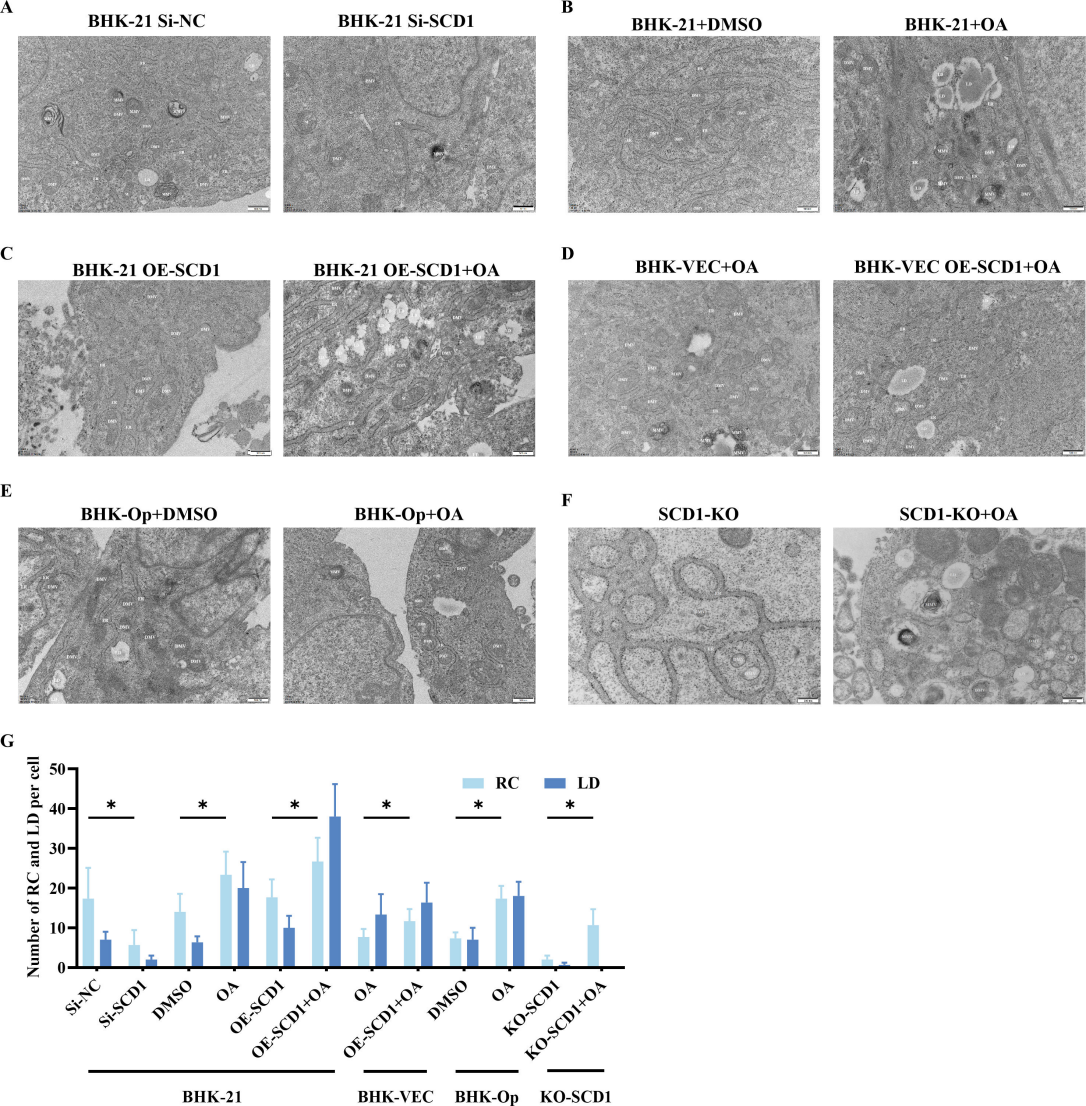
SCD1 and its enzyme products are involved in FMDV replication complex formation. (A–F) Transmission electron microscopy was performed as described in Materials and Methods. In the figures, “M” indicates mitochondria, “ER” indicates the endoplasmic reticulum, “DMV” indicates double-membrane vesicles, and “LD” indicates lipid droplets. Scale bars, 200 nm and 500 nm. (**G**) The number of replication complexes (RCs) and lipid droplets (LDs) in five random cells. *n* = 3; *P < 0.05; n.s., not significant.

To further investigate the role of SCD1 in the FMDV replication complex, we observed replication complexes in BHK-21, BHK-VEC, and BHK-Op cells by TEM. We also observed the number and morphology of replication complexes in *SCD1*-overexpressing or *SCD1*-knockdown BHK-21 and BHK-VEC cells to study the effect of SCD1 on the establishment of replication complexes. The numbers of both replication complexes, and lipid droplets were greatly reduced in cells with *SCD1* knockdown compared with cells without SCD1 knockdown (Fig. 5A and G). After the addition of exogenous OA, the number of lipid droplets and FMDV replication complexes in the cells significantly increased (Fig. 5B and G). This difference was more pronounced in BHK-Op cells than in the other cell lines. When *SCD1* was overexpressed, the number of replication complexes and lipid droplets significantly increased (Fig. 5C and G).

We did not observe replication complexes in FMDV-infected BHK-VEC cells (data not shown), which may explain why FMDV cannot replicate in BHK-VEC cells. However, after the addition of exogenous OA or expression of SCD1, replication complexes and lipid droplets formed in BHK-VEC cells (Fig. 5D and G). Multiple lamellar vesicles were produced simultaneously, but their number was markedly lower and they were more dispersed than those in BHK-21 cells. This is consistent with our previous finding that overexpression of SCD1 or exogenous OA could not completely restore FMDV replication in BHK-VEC cells. The addition of OA to BHK-Op cells resulted in increased replication complexes and lipid droplets (Fig. 5E). The addition of exogenous OA to *SCD1*-KO cells increased the number of intracellular lipid droplets and replication complexes (Fig. 5F and G). These results demonstrated that upregulation of SCD1 activity led to endoplasmic reticulum rupture in host cells, which in turn promoted FMDV replication complex formation.

In conclusion, the upregulation of SCD1 activity leads to an increase in the number of replication complexes and lipid droplets. In contrast, downregulation of SCD1 activity decreases the number of replication complexes and lipid droplets and also leads to abnormal FMDV replication complex morphology. These findings suggest that SCD1 is a host cell factor required for the formation of replication complexes by FMDV.

### The replication of other RNA viruses is regulated by SCD1 enzyme activity

**Fig 6:**
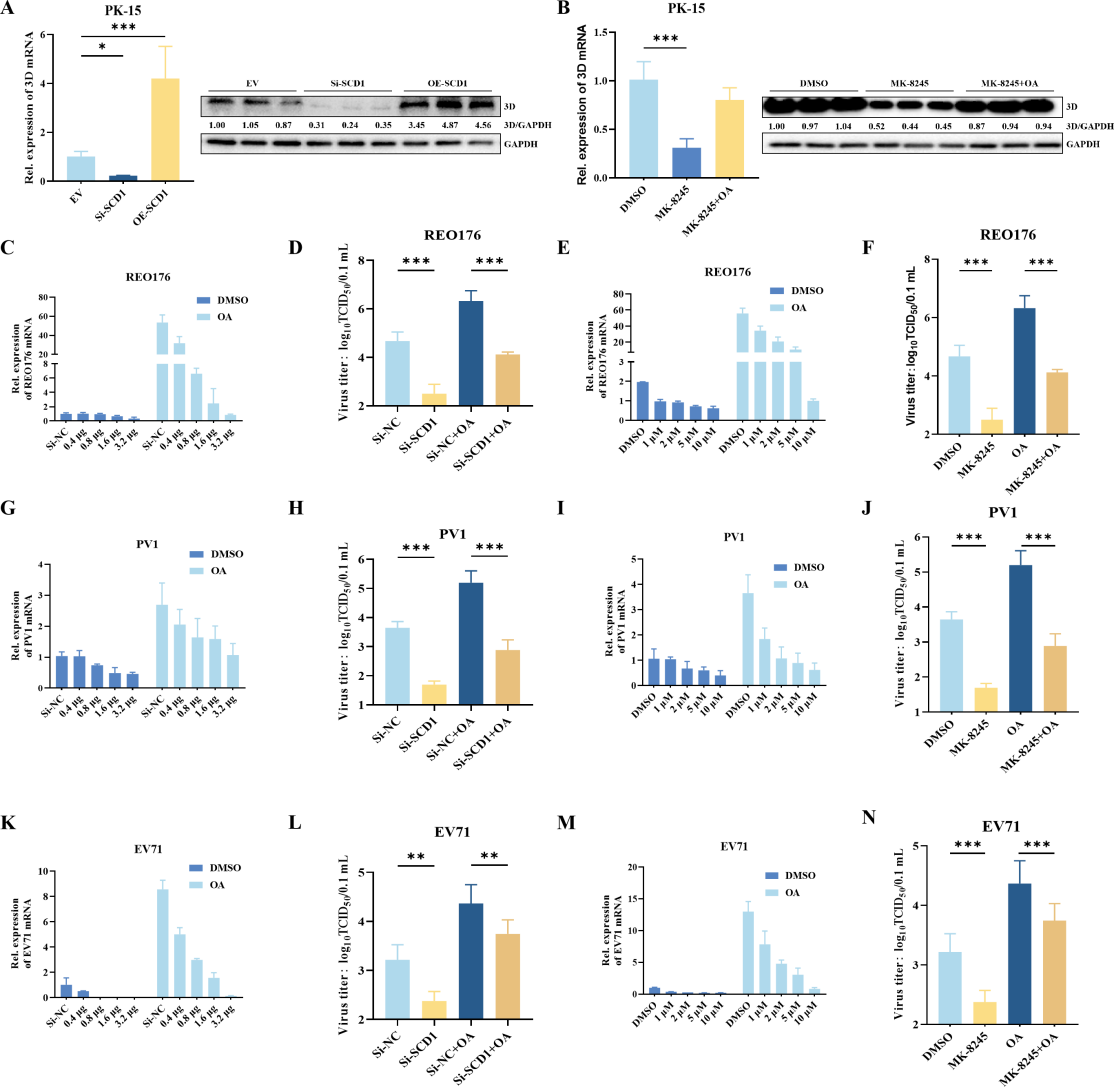
A broad spectrum of positive-strand RNA virus replication is regulated by SCD1 enzyme activity. (**A**) PK-15 (pig kidney epithelial) cells were transfected with OE-SCD1 or Si-SCD1, and at 24 hpt, the cells were infected with FMDV. After 16 h of culture, the expression levels of FMDV 3D protein and vRNA were determined. (**B**) FMDV-infected PK-15 cells were cultured in media supplemented with an SCD1 inhibitor with or without OA. Cells were collected at 16 hpi for analysis of 3D expression. (C and D) Respiratory enteric orphan virus-3-176 (REO176)-infected Vero cells were cultured in 2% minimal essential medium for 3 h. The cells were collected after an additional 16 h of culture for vRNA detection (**C**); the culture supernatant was collected for virus titration by the TCID50 method (**D**). (E and F) REO176-infected Vero cells were cultured in a medium containing different concentrations of MK8245, and the cells were collected at 16 hpi for vRNA detection (**E**); or the culture supernatant was collected for virus titration (**F**). (**G**) PV1-infected Vero cells were cultured in media containing different concentrations of OA. After 16 h of culture, the cells were collected for vRNA detection. (**H**) PV1-infected Vero cells were cultured in media containing MK-8245, OA, or MK-8245 + OA. After 16 h of culture, the culture supernatant was collected for virus titration. (**I**) Vero cells were infected with PV1, and after 3 h the medium was replaced with a maintenance medium containing different concentrations of MK8245. After 16 h, RNA was extracted from the cells and reverse transcribed into cDNA, and the RNA level of PV1 was detected by qPCR. (**J**) Vero cells were infected with PV1 and, after 3 h, the medium was replaced with a maintenance medium containing different concentrations of MK8245. The supernatant was collected after an additional 16 h of culture, and the viral titer in the supernatant was determined by the TCID50 method. (**K**) EV71-infected Vero cells were cultured in media containing different concentrations of OA. After 16 h of culture, the cells were collected for viral RNA detection. (**L**) EV71-infected Vero cells were cultured in media containing MK-8245, OA, or MK-8245 + OA. After 16 h of culture, the supernatant was collected for virus titration. (**M**) Vero cells were infected with EV71 for 3 h, after which the medium was replaced with a maintenance medium containing different concentrations of MK8245. After 16 h of culture, the cells were collected for EV71 RNA detection by qPCR. (**N**) Vero cells were infected with EV71, and after 3 h the medium was replaced with a maintenance medium containing different concentrations of MK8245. The supernatant was collected 16 h later, and the virus titer was determined by the TCID50 method. *n* = 3; *P < 0.05; n.s., not significant.

To explore whether SCD1 regulates FMDV replication in cells from other species, we knocked down *SCD1* expression using RNAi or inhibited its enzyme activity using SCD1 inhibitors in PK-15 cells (a pig kidney epithelial cell line) and observed a significant decrease in FMDV replication efficiency in PK-15 cells (Fig. 6A and B).

To evaluate the effect of SCD1-regulated lipid metabolism on the replication of other positive-sense RNA viruses, we used the positive-stranded RNA viruses PV1 and EV71; we also tested the double-stranded RNA virus REO176, and the negative-stranded RNA virus VSV. In Vero cells transfected with Si-SCD1, REO176 replication was inhibited (as determined by RT-qPCR) (Fig. 6C). After chemically inhibiting the activity of SCD1, we added 200 µM OA; the decrease in the replication level of REO176 was reversed (Fig. 6C). This result was confirmed by measuring viral TCID_50_ of viral titers (Fig. 6D). We then added different concentrations of the SCD1 inhibitor to Vero cells, followed by supplementation with OA. Consistent with the *SCD1* knockdown, the exogenous SCD1 inhibitor inhibited REO176 replication, while supplementation with OA reversed this inhibition (Fig. 6E). Inhibition of SCD1 activity decreased REO176 titers, whereas supplementation with OA upregulated REO176 titers (Fig. 6F). Similar results were observed for the positive-sense RNA viruses PV1 (Fig. 6G through J), and EV71 (Fig. 6K through N). To further investigate whether SCD1 is a host cell factor necessary for the replication of negative-strand RNA viruses, we knocked down SCD1 in Vero cells and detected the mRNA and titer of VSV viruses. Both the mRNA level and titer of VSV were significantly decreased after SCD1 was knocked down in Vero cells (Fig. S4), and thus SCD1 is also involved in negative-strand RNA virus replication. Thus, SCD1 is essential for FMDV replication in a variety of cells from different mammalian species. Meanwhile, SCD1 is involved in regulating the replication of various types of RNA viruses.

## DISCUSSION

FMDV replication is closely related to host cell lipid metabolism, and SCD1 is a rate-limiting enzyme in the biosynthesis of monounsaturated fatty acids from saturated fatty acid precursors that catalyzes the production of the monounsaturated fatty acid OA from stearic acid. By regulating SCD1 expression, we were able to influence FMDV replication in BHK-21, BHK-VEC, and BHK-Op cells. The addition of either the catalytic product of SCD1 (i.e., OA) or its substrate, SA, to these cells, promoted FMDV replication. Interestingly, OA addition or SCD1 overexpression in BHK-VEC cells, in which SCD1 expression is usually low, is activated in FMDV replication. When we exogenously added moderate amounts of OA to FMDV-infected cells in which SCD1 was knocked down or knocked out, FMDV replication was restored to normal levels. Our results also showed that the SCD1 enzymatic activity, to produce OA, is controlled by the AMP-activated protein kinase (AMPK) pathway and that promotion of AMPK pathway signaling inhibited FMDV replication (Fig. S5). Although the *SCD1* gene, an important host factor affecting FMDV replication, was involved in the establishment of the BHK-Op and BHK-VEC cell models, the establishment of the model was the result of the coordination of multiple genes.

To investigate the effect of SCD1 catalytic activity on FMDV replication, we constructed a triple-histidine point mutant of SCD1 and found that the inactivation of SCD1 enzyme activity led to significant downregulation of FMDV replication. The binding of SCD1 to FMDV protein 2C may play a role in assisting FMDV replication, but the exact mechanism through which this occurs is still unclear. IF experiments confirmed the co-localization of SCD1 with 2C in the replication complex. Interestingly, SCD1, an endoplasmic reticulum-anchored protein, is not normally found in other subcellular structures. However, after infection with FMDV, the host cell undergoes membrane rearrangement, and this membrane rearrangement likely leads to the entry of SCD1 into the bilayer of the replication complex and its co-localization with 2C. In addition, 2C is involved in and mediates membrane rearrangements in host cells of FMDV, while the SCD1 product OA contributes to generating negative membrane curvature and provides the raw material for the replication complex. Together, these factors complement each other to promote the establishment of the FMDV replication complex (25, 26). However, the detailed mechanism through which SCD1 functions in this context needs to be further investigated. We extracted detergent-resistant membrane structures from cells and found that the level of 2C in detergent-resistant membranes was significantly lower in *SCD1*-knockdown cells than in normal BHK-21 cells. In HCV, the proteins involved in replication complexes have been shown to be highly detergent-resistant (6), suggesting that the synthesis of HCV RNA occurs on lipid raft membrane structures. HCV nonstructural proteins exhibit strong detergent resistance and are present on the lipid rafts of nonstructural protein NS3-5B overexpressing cells (27). This result that FMDV shares the same replicative form as HCV and that both nonstructural proteins and host cytokines require lipid raft structures to assist viral replication.

We also performed IF experiments and found that *SCD1* knockdown shifted the subcellular localization of 2C from the perinuclear region, where the FMDV replication complex is located, to the cytoplasm. These results suggest that the decrease in SCD1 activity leads to a change in 2C localization and a decrease in the amount of 2C in the detergent-resistant membranes of replication complexes. However, it is worth noting that both our previous demonstration of the role of SCD1 enzymatic activity and validation of the 2C recruitment function of SCD1 revealed that there are other factors in host cells that can recruit nonstructural FMDV proteins and that SCD1 is not the only host factor that is important for the establishment of the FMDV replication complex. The discovery of other host factors with similar abilities to recruit nonstructural proteins or promote the establishment of the FMDV replication complex is one of the main directions of our future work.

A common feature of positive-strand RNA viruses is that they cause remodeling of the inner membrane of infected cells. To undergo replication, positive-strand RNA requires a membrane spacer, in which dsRNA, a marker of the replication complex, is produced before viral assembly (28). The membrane source of bilayer vesicles, the replication complex, may also contain other membrane structures, such as lipid droplets, mitochondria, and early and late endosomes (29). Our results indicate that the replication complex is located near the endoplasmic reticulum, and we observed multilayered vesicles and bilayered vesicles. Such multilayered vesicles are thought to occur in the late stages of HCV infection and are not associated with RNA replication. In addition, the inhibition of SCD1 activity resulted in structural abnormalities and a decrease in the number of replication complexes. Conversely, overexpression of SCD1 or the addition of OA increased the number of replication complexes, thus facilitating viral replication. Notably, we successfully reconstructed the replication complex in BHK-VEC cells by upregulating SCD1 activity, suggesting that abnormal formation of the replication complex is one of the important reasons why FMDV fails to replicate properly in BHK-VEC cells. However, the structure of the replication complex in these cells was still abnormal after SCD1 overexpression, suggesting that there are other host factors involved in the formation of the replication complex. The above evidence strongly suggests that SCD1 regulates host cell lipid metabolism and participates in replication complex formation, which in turn regulates FMDV replication. Additionally, SCD1 promoted the collapse of the endoplasmic reticulum after FMDV infection of host cells, thereby promoting the formation of replication complexes. This finding provides key evidence that SCD1 is directly involved in the formation of the FMDV replication complex. Taken together with the results of previous studies, our findings suggest that SCD1 is involved in recruiting FMDV protein 2C to the replication complex. Therefore, disruption of SCD1 activity can decrease the 2C content in the replication complex.

To investigate the role of SCD1-mediated lipid metabolism in promoting the replication of RNA viruses in general, we inhibited SCD1 activity and observed the regulation of REO176, PV1, EV71, and VSV replication. Inhibition of SCD1 activity downregulated the replication of each of these viruses, while the addition of exogenous OA reversed this inhibition. This finding suggests that SCD1 also regulates the replication of these RNA viruses by modulating lipid metabolism and thus the formation of the viral replication complexes. Our results demonstrated that the knockdown of *SCD1* does not affect cellular activity and that inhibition of SCD1 activity inhibits RNA replication. Therefore, we believe that SCD1 is a potential target for the treatment of infections by RNA viruses. Our findings provide new ideas for the development of antiviral drugs.

## Data Availability

The data that support the findings of this study are available from the corresponding author upon reasonable request.

## SUPPLEMENTAL MATERIAL

Supplemental figures jvi.00902-24-s0001.doc Figures S1 to S5.

ASM does not own the copyrights to Supplemental Material that may be linked to, or accessed through, an article. The authors have granted ASM a non-exclusive, world-wide license to publish the Supplemental Material files. Please contact the corresponding author directly for reuse.

## References

[1] Zhang Z, He G, Filipowicz NA, Randall G, Belov GA, Kopek BG, Wang X. 2019. Host lipids in positive-strand RNA virus genome replication. Front Microbiol 10:286. doi:10.3389/fmicb.2019.00286 30863375 PMC6399474

[2] Li X, Wang M, Cheng A, Wen X, Ou X, Mao S, Gao Q, Sun D, Jia R, Yang Q, Wu Y, Zhu D, Zhao X, Chen S, Liu M, Zhang S, Liu Y, Yu Y, Zhang L, Tian B, Pan L, Chen X. 2020. Enterovirus replication organelles and inhibitors of their formation. Front Microbiol 11:1817. doi:10.3389/fmicb.2020.01817 32973693 PMC7468505

[3] Perera R, Riley C, Isaac G, Hopf-Jannasch AS, Moore RJ, Weitz KW, Pasa-Tolic L, Metz TO, Adamec J, Kuhn RJ. 2012. Dengue virus infection perturbs lipid homeostasis in infected mosquito cells. PLoS Pathog 8:e1002584. doi:10.1371/journal.ppat.1002584 22457619 PMC3310792

[4] Heaton NS, Perera R, Berger KL, Khadka S, Lacount DJ, Kuhn RJ, Randall G. 2010. Dengue virus nonstructural protein 3 redistributes fatty acid synthase to sites of viral replication and increases cellular fatty acid synthesis. Proc Natl Acad Sci USA 107:17345–17350. doi:10.1073/pnas.1010811107 20855599 PMC2951450

[5] Mohamed B, Mazeaud C, Baril M, Poirier D, Sow AA, Chatel-Chaix L, Titorenko V, Lamarre D. 2020. Very-long-chain fatty acid metabolic capacity of 17-beta-hydroxysteroid dehydrogenase type 12 (HSD17B12) promotes replication of hepatitis C virus and related flaviviruses. Sci Rep 10:4040. doi:10.1038/s41598-020-61051-w 32132633 PMC7055353

[6] Nguyen LN, Lim Y-S, Pham LV, Shin H-Y, Kim Y-S, Hwang SB. 2014. Stearoyl coenzyme A desaturase 1 is associated with hepatitis C virus replication complex and regulates viral replication. J Virol 88:12311–12325. doi:10.1128/JVI.01678-14 25122791 PMC4248942

[7] Midgley R, Moffat K, Berryman S, Hawes P, Simpson J, Fullen D, Stephens DJ, Burman A, Jackson T. 2013. A role for endoplasmic reticulum exit sites in foot-and-mouth disease virus infection. J Gen Virol 94:2636–2646. doi:10.1099/vir.0.055442-0 23963534 PMC3836498

[8] Wileman T. 2007. Aggresomes and pericentriolar sites of virus assembly: cellular defense or viral design? Annu Rev Microbiol 61:149–167. doi:10.1146/annurev.micro.57.030502.090836 17896875

[9] Delang L, Harak C, Benkheil M, Khan H, Leyssen P, Andrews M, Lohmann V, Neyts J. 2018. PI4KIII inhibitor enviroxime impedes the replication of the hepatitis C virus by inhibiting PI3 kinases. J Antimicrob Chemother 73:3375–3384. doi:10.1093/jac/dky327 30219827

[10] Brown JM, Chung S, Sawyer JK, Degirolamo C, Alger HM, Nguyen T, Zhu X, Duong M-N, Wibley AL, Shah R, Davis MA, Kelley K, Wilson MD, Kent C, Parks JS, Rudel LL. 2008. Inhibition of stearoyl-coenzyme A desaturase 1 dissociates insulin resistance and obesity from atherosclerosis. Circulation 118:1467–1475. doi:10.1161/CIRCULATIONAHA.108.793182 18794388 PMC2716169

[11] Nakamura MT, Nara TY. 2004. Structure, function, and dietary regulation of delta6, delta5, and delta9 desaturases. Annu Rev Nutr 24:345–376. doi:10.1146/annurev.nutr.24.121803.063211 15189125

[12] Bai Y, McCoy JG, Levin EJ, Sobrado P, Rajashankar KR, Fox BG, Zhou M. 2015. X-ray structure of a mammalian stearoyl-CoA desaturase. Nature 524:252–256. doi:10.1038/nature14549 26098370 PMC4689147

[13] Wang H, Klein MG, Zou H, Lane W, Snell G, Levin I, Li K, Sang B-C. 2015. Crystal structure of human stearoyl-coenzyme A desaturase in complex with substrate. Nat Struct Mol Biol 22:581–585. doi:10.1038/nsmb.3049 26098317

[14] Lyn RK, Singaravelu R, Kargman S, O’Hara S, Chan H, Oballa R, Huang Z, Jones DM, Ridsdale A, Russell RS, Partridge AW, Pezacki JP. 2014. Stearoyl-CoA desaturase inhibition blocks formation of hepatitis C virus-induced specialized membranes. Sci Rep 4:4549. doi:10.1038/srep04549 25008545 PMC4091094

[15] Jin P, Bo L, Liu Y, Lu W, Lin S, Bian J, Deng X. 2016. Activator protein 1 promotes the transcriptional activation of IRAK-M. Biomed Pharmacother 83:1212–1219. doi:10.1016/j.biopha.2016.08.024 27562721

[16] Kobori H, Hayashi M, Saruta T. 2001. Thyroid hormone stimulates renin gene expression through the thyroid hormone response element. Hypertension 37:99–104. doi:10.1161/01.hyp.37.1.99 11208763 PMC2573046

[17] Scaglia N, Chisholm JW, Igal RA. 2009. Inhibition of stearoylCoA desaturase-1 inactivates acetyl-CoA carboxylase and impairs proliferation in cancer cells: role of AMPK. PLoS One 4:e6812. doi:10.1371/journal.pone.0006812 19710915 PMC2728543

[18] Parker KA, Ribet S, Kimmel BR, Dos Reis R, Mrksich M, Dravid VP. 2022. Scanning transmission electron microscopy in a scanning electron microscope for the high-throughput imaging of biological assemblies. Biomacromolecules 23:3235–3242. doi:10.1021/acs.biomac.2c00323 35881504

[19] Tan S-H, Shui G, Zhou J, Shi Y, Huang J, Xia D, Wenk MR, Shen H-M. 2014. Critical role of SCD1 in autophagy regulation via lipogenesis and lipid rafts-coupled AKT-FOXO1 signaling pathway. Autophagy 10:226–242. doi:10.4161/auto.27003 24296537 PMC5396079

[20] Yuan Y, Wang X, Li J, Han L, Du H, Sun Y, Yang P, Zhou Z, Gu M, Lu Y, Shen C. 2022. Single-cell sequencing yields insights in the evolution of foot-and-mouth disease virus persistent infection. Front Cell Infect Microbiol 12:940906. doi:10.3389/fcimb.2022.940906 35873170 PMC9304859

[21] Han L, Yuan Y, Hu J, Li J, Zhu S, Yang P, Cheng A, Li X, Shen C. 2021. Next-generation sequencing sheds light on the interaction between virus and cell during foot-and-mouth disease virus persistent infection. Vet Microbiol 263:109247. doi:10.1016/j.vetmic.2021.109247 34649012

[22] Lou Y, Shanklin J. 2010. Evidence that the yeast desaturase Ole1p exists as a dimer in vivo. J Biol Chem 285:19384–19390. doi:10.1074/jbc.M110.125377 20406812 PMC2885218

[23] Shanklin J, Whittle E, Fox BG. 1994. Eight histidine residues are catalytically essential in a membrane-associated iron enzyme, stearoyl-CoA desaturase, and are conserved in alkane hydroxylase and xylene monooxygenase. Biochemistry 33:12787–12794. doi:10.1021/bi00209a009 7947684

[24] Zhang C, Yang F, Wojdyla JA, Qin B, Zhang W, Zheng M, Cao W, Wang M, Gao X, Zheng H, Cui S. 2022. An anti-picornaviral strategy based on the crystal structure of foot-and-mouth disease virus 2C protein. Cell Rep 40:111030. doi:10.1016/j.celrep.2022.111030 35793627

[25] Oberhauser L, Granziera S, Colom A, Goujon A, Lavallard V, Matile S, Roux A, Brun T, Maechler P. 2020. Palmitate and oleate modify membrane fluidity and kinase activities of INS-1E β-cells alongside altered metabolism-secretion coupling. Biochim Biophys Acta Mol Cell Res 1867:118619. doi:10.1016/j.bbamcr.2019.118619 31816355

[26] Reglinski K, Steinfort-Effelsberg L, Sezgin E, Klose C, Platta HW, Girzalsky W, Eggeling C, Erdmann R. 2020. Fluidity and lipid composition of membranes of peroxisomes, mitochondria and the ER from oleic acid-induced Saccharomyces cerevisiae. Front Cell Dev Biol 8:574363. doi:10.3389/fcell.2020.574363 33195209 PMC7658010

[27] Gao L, Aizaki H, He J-W, Lai MMC. 2004. Interactions between viral nonstructural proteins and host protein hVAP-33 mediate the formation of hepatitis C virus RNA replication complex on lipid raft. J Virol 78:3480–3488. doi:10.1128/jvi.78.7.3480-3488.2004 15016871 PMC371042

[28] Miller S, Krijnse-Locker J. 2008. Modification of intracellular membrane structures for virus replication. Nat Rev Microbiol 6:363–374. doi:10.1038/nrmicro1890 18414501 PMC7096853

[29] Romero-Brey I, Merz A, Chiramel A, Lee J-Y, Chlanda P, Haselman U, Santarella-Mellwig R, Habermann A, Hoppe S, Kallis S, Walther P, Antony C, Krijnse-Locker J, Bartenschlager R. 2012. Three-dimensional architecture and biogenesis of membrane structures associated with hepatitis C virus replication. PLoS Pathog 8:e1003056. doi:10.1371/journal.ppat.1003056 23236278 PMC3516559

