# Supplementary material for: Stearoyl coenzyme A desaturase 1 (SCD1) regulates foot-and-mouth disease virus replication by modulating host cell lipid metabolism and viral protein 2C-mediated replication complex formation
Source: J Virol. 2024 Sep 26;98(10):e00902-24. doi: 10.1128/jvi.00902-24 (PMC11495015; doi:10.1128/jvi.00902-24)
Supplement: Supplemental figures — Figures S1 to S5. [file jvi.00902-24-s0001.doc]

**Stearoyl coenzyme A desaturase 1 (SCD1) regulates foot-and-mouth disease virus replication by modulating host cell lipid metabolism and 2C-mediated replication complex formation**

Bonan Lv^1,2^, Yuncong Yuan^1,2^, Zhuang Yang^1,2^, Xingran Wang^1,2^, Jianjun Hu^1,2^, Yidan Sun^1,2^, Hang Du^1,2^, Xuemei Liu^1,2^, Huimin Duan^1,2^, Ruyi Ding^1,2^, Zishu Pan^2^, Xiao-Feng Tang^1*^, Chao Shen^1,2*^

1 Hubei Key Laboratory of Cell Homeostasis, College of Life Sciences, Wuhan University，Wuhan 430072, China

2 State Key Laboratory of Virology, College of Life Sciences, Wuhan University, Wuhan 430072, China

Corresponding author: Chao Shen [(shenchao@whu.edu.cn)](mailto:(shenchao@whu.edu.cn)), Xiao-Feng [Tang(tangxf@whu.edu.cn)](mailto:Tang(tangxf@whu.edu.cn),)

**Supplemental information**


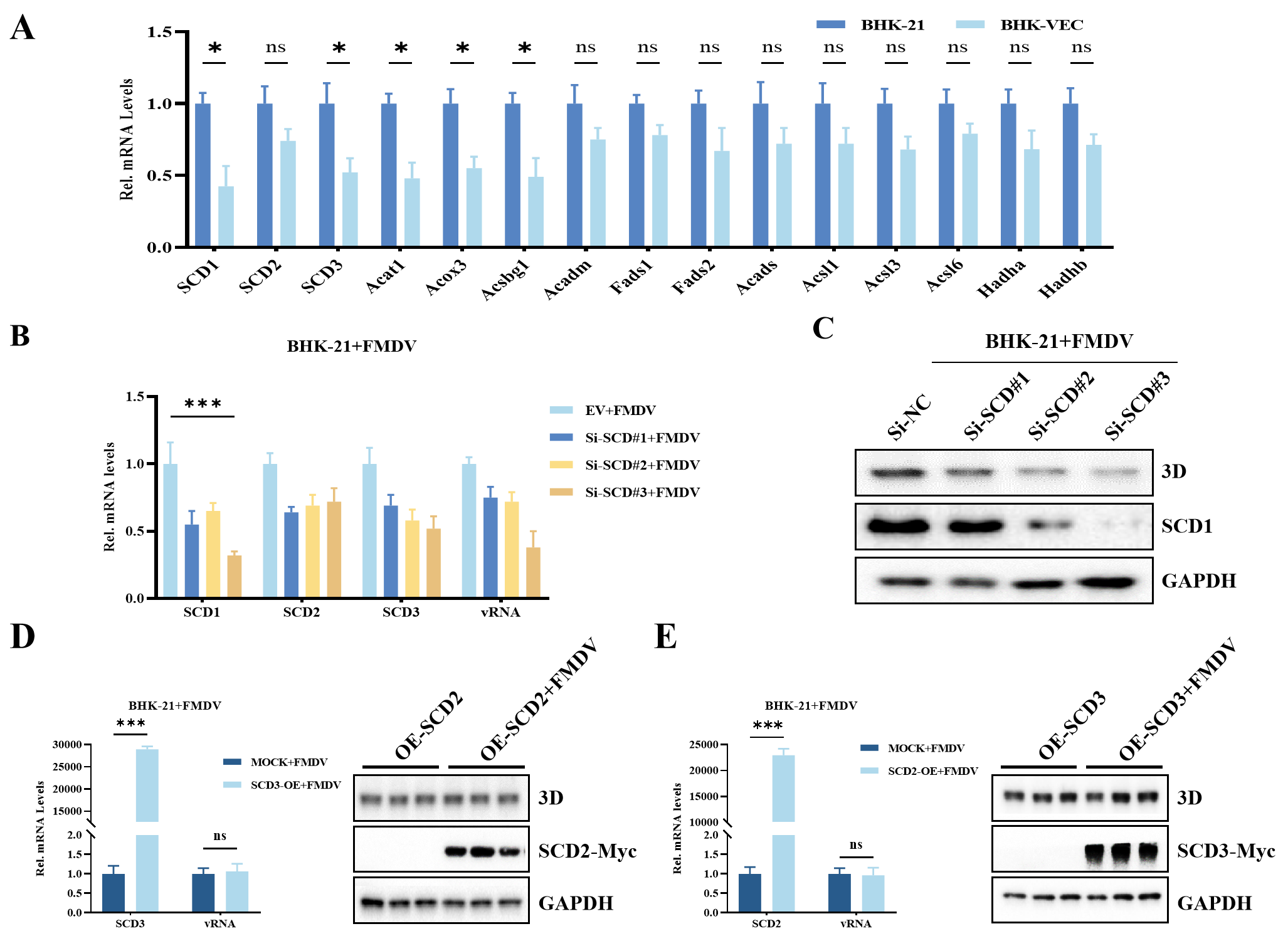


**Figure S1 Functional characterization of the *SCD1* gene**

(A) Expression analysis of genes enriched in the fatty acid metabolism pathway via transcriptome sequencing of BHK-VEC cells and BHK-21 cells; (B) BHK-21 cells transfected with the SCD gene RNAi vector were infected with foot-and-mouth disease virus (FMDV) at 16 hours post transfection (hpt). After 24 h of culture, the infected cells were harvested for SCD gene or 3D detection via qPCR. (C) BHK-21 cells transfected with the SCD gene RNAi vector were transfected with FMDV at 16 (hpt). After 24 h of culture, the infected cells were collected and subjected to immunoblotting to detect the protein levels of 3D and SCD1. (D&E) BHK-21 cells were transiently transfected with SCD2 or SCD3 overexpression vectors, respectively, and infected with FMDV for 16 h after 24 h of transfection to detect the levels of SCD2, SCD3, and vRNA, as well as the levels of SCD2, SCD3, and 3D protein. n=3. **P* < 0.05, n.s., not significant.


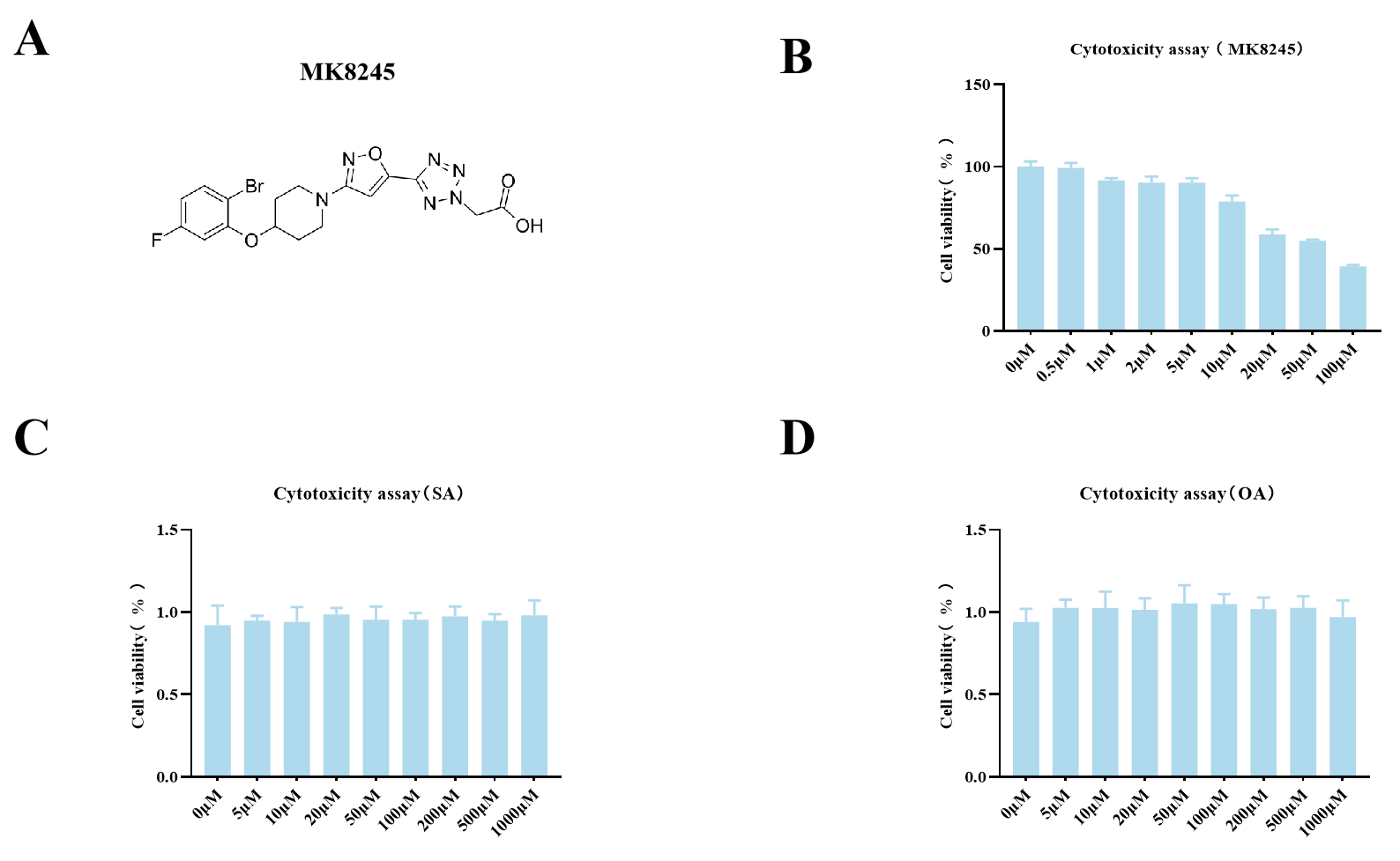


**Figure S2 MK-8245, OA and SA cytotoxicity assay**

(A)MK-8245 Chemical Structure; (B&C&D) Different concentrations of MK-8245, OA and SA were added to each well in 96-well plates, and BHK-21 cells were treated for 2 days, and the cell viability was determined by CCK8 assay (n=8).


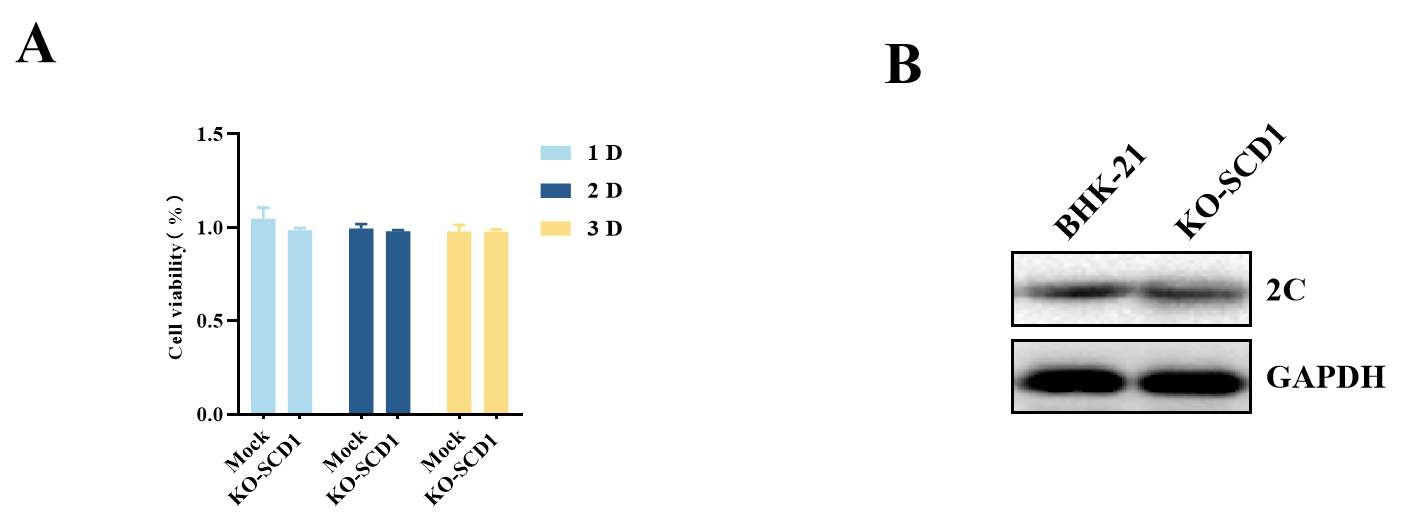


**Figure S3 Effect of SCD1 knockdown on BHK-21 cell activity and FMDV 2C protein expression**

(A)Normal BHK-21 cells and *SCD1*-KO cells were cultured separately for 3 day (D), and cell numbers were measured every 24 h. (B) BHK-21 and *SCD1*-KO cells were infected with FMDV, and cells were infected with FMDV and harvested for 2C protein assay after 16 h of culture. n=3.


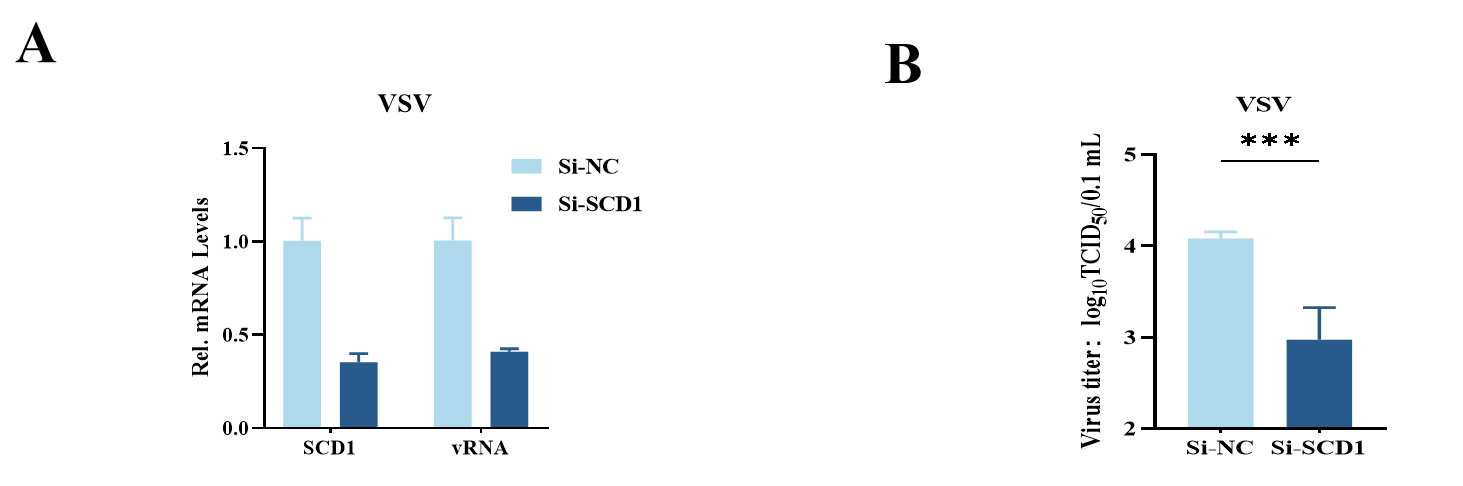


**Figure S4 VSV replication is regulated by SCD1 enzyme activity**

(A&B) Vero cells were transfected with Si-SCD1 or Si-NC and at 24 hpt cells infected with VSV. Cells were collected for vRNA detection after an additional 16 h of incubation (A); Cells culture supernatants were collected at and titrated for virus using the TCID_50_ (B) method. n=3. **P* < 0.05, n.s., not significant.


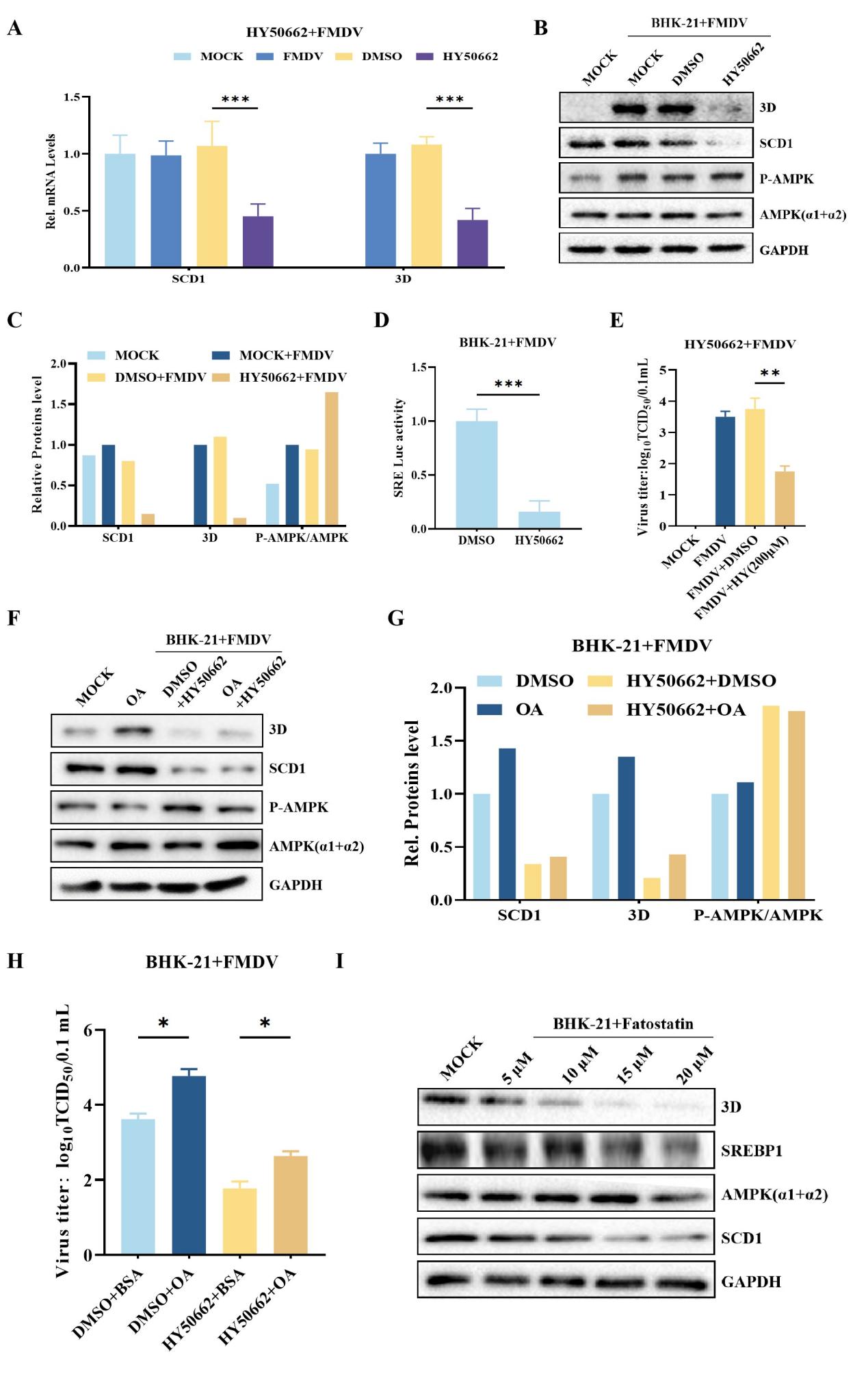


**Figure S5 SCD1-mediated regulation of FMDV replication is dependent on the AMPK pathway**

1. FMDV-infected BHK-21 cells were cultured in media supplemented with HY50662 for 16 h, and infected cells were collected for detection of SCD1 or vRNA levels via qPCR or (B) for detection of AMPK, p-AMPK, SCD1, or 3D protein. (C) Image-pro Plus 6.0 software was used to quantify the gray values of the protein bands from the western blot in Figure C, after which the gray values of the target proteins were compared to those of the corresponding internal references. (D) FMDV-infected BHK-21 cells were cultured in medium containing HY50662. After 16 h of culture, the cells were collected and lysed for transcriptional activation level detection using a luciferase reporter or (E) for determination of the viral titer in the supernatant using the TCID_50_ method. (F) FMDV-infected BHK-21 cells were cultured in medium containing HY50662, after which the BHK-21 cells were treated with different concentrations of OA. After 16 h of culture, the cells were subjected to AMPK, p-AMPK, SCD1, or 3D protein detection. (G) Image-Pro Plus 6.0 software was used to analyze the protein bands of the western blot in Figure C. The protein bands of the western blot were quantified by gray value, and the gray value of the target protein was subsequently compared to the gray value of the corresponding internal reference. (H) FMDV-infected BHK-21 cells were cultured in medium containing HY0662 and different concentrations of OA. The supernatant was collected at 16 h postinfection for viral titration using the TCID_50_ method. (I) FMDV-infected BHK-21 cells were cultured in Fatostain-containing medium. After 16 h of culture, the infected cells were harvested for SREBP1, AMPK, SCD1, or 3D protein detection. (n=3 for each group of experiments; *p<0.05, **p<0.01, n.s. not significantly different)
